# Astragaloside IV modulates oxidative stress and osteoimmune–Wnt signaling in ovariectomized rats: an integrated study of RNA sequencing, molecular docking, and experimental validation

**DOI:** 10.3389/fnut.2026.1785452

**Published:** 2026-04-13

**Authors:** Ya-Qing Li, Xian-Ying Yao, Yan Jing, Zi-Yi Liu, Xing-Wang Wang, Ming Liang, Shuai-Yu Jiang, Tao Lu, Chen Chen, Yan-Ping Gao

**Affiliations:** 1 First Clinical College, Shanxi University of Traditional Chinese Medicine, Jinzhong, China; 2 Clinical Research Laboratory, Shanxi Province Key Laboratory Cultivation Base Jointly Established by the Department and City of Hormone Metabolic Diseases During Perimenopause, The First People's Hospital of Datong, Datong, China; 3 Changzhi Medical College, Changzhi, China; 4 Datong Key Laboratory of Smart Medicine and Health Care for Elderly Chronic Diseases, Shanxi Datong University, Datong, China; 5 Yueyang Clinical Medical College, Shanghai University of Traditional Chinese Medicine, Shanghai, China

**Keywords:** astragaloside IV, bone remodeling, histopathology, micro-computed tomography, molecular docking, ovariectomized rats, postmenopausal osteoporosis, RNA sequencing

## Abstract

**Background:**

Postmenopausal osteoporosis (PMOP) is characterized by high-turnover bone loss and oxidative stress. Astragaloside IV (AS-IV) exhibits antioxidant and immunomodulatory activities, yet its skeletal effects and mechanistic basis in ovariectomized (OVX) models remain incompletely defined.

**Methods:**

Female Sprague–Dawley rats underwent bilateral ovariectomy. Six weeks after surgery, OVX rats were randomized to model, positive control, or AS-IV treatment (20/40/80 mg/kg, gavage, 8 weeks), with sham-operated controls. Serum bone-turnover markers (CTX-I, OCN, PTH) and oxidative stress markers (SOD, MDA) were measured by ELISA. Femora were assessed by micro-CT (BMD, BV/TV, Tb.N, Tb.Sp) and H&E staining. RNA sequencing was performed followed by GO/KEGG enrichment and WGCNA to identify hub genes, and molecular docking was used to evaluate AS-IV–target interactions. Multiplex immunofluorescence in the femoral trabecular bone quantified Lep, Ptgs2, Gfap, Igfbp2, Wnt1, *β*-catenin, and NF-κB p65 region (mean fluorescence intensity, normalized to Sham).

**Results:**

OVX rats developed a clear high-turnover phenotype with trabecular rarefaction, reflected by higher CTX-I and MDA and lower SOD. AS-IV produced a dose-related improvement in these readouts—CTX-I fell, oxidative stress was eased (SOD increased and MDA decreased), and OCN and PTH moved back toward control levels. Histological analysis showed AS-IV treatment partially mitigated the trabecular deterioration observed in OVX rats. However, micro-CT analysis showed no statistically significant differences in trabecular bone structure between AS-IV-treated groups and OVX controls (*p* > 0.05). RNA-seq identified osteoimmune and Wnt-centered programs and nominated hub genes including Lep, Ptgs2, Gfap, Igfbp2, Il22ra2, Wnt10a, and Wnt1. In femoral trabecular bone, multiplex immunofluorescence revealed increased NF-κB p65 and decreased Wnt1 and Gfap signals in OVX rats compared to Sham. High-dose AS-IV significantly reduced NF-κB p65 and partly restored Wnt1 and Gfap. However, Ptgs2 showed no significant differences across groups, and Igfbp2 and *β*-catenin were not significantly rescued within this sampling window. Molecular docking suggested favorable binding between AS-IV and selected targets, supporting its multitarget profile.

**Conclusion:**

AS-IV modulates oxidative stress and osteoimmune–Wnt signaling in OVX rats, with directional improvements in bone turnover markers but without significant micro-CT structural changes. This work provides a foundation for hypothesis-driven investigation of AS-IV in PMOP.

## 1 Introduction

Postmenopausal osteoporosis (PMOP) is a metabolic bone disease characterized by decreased bone mass and structural destruction of bone tissue due to estrogen deficiency, leading to enhanced bone fragility and fracture risk (1, 2). The incidence of fractures varies greatly by country, but on average up to 50% of women >50 years of age are at risk of fractures (2). With the acceleration of global ageing, PMOP poses a serious challenge to the health and quality of life of hundreds of millions of middle-aged and older women around the world, as well as an enormous socio-economic burden (3). The core pathological mechanism of PMOP is a significant increase in bone remodeling activity of estrogen-deficient osteoclasts (4) and a relative lack of bone formation by osteoblasts, leading to an imbalance in bone reconstruction (5).

Currently, hormone replacement therapy (HRT) serves as a direct intervention for estrogen deficiency (6). However, its long-term application is associated with increased risk of breast cancer (7), endometrial cancer (8), and coronary heart disease (9), stroke, thromboembolic events with 5 or more years of use (10) limiting its clinical application. Furthermore, the presence of various contraindications, including hypertension, diabetes, and a family history of breast cancer, further restricts its use in eligible patients (11, 12). These shortcomings have shifted attention to safer, more effective options—especially those derived from natural products.

**Figure 1:**
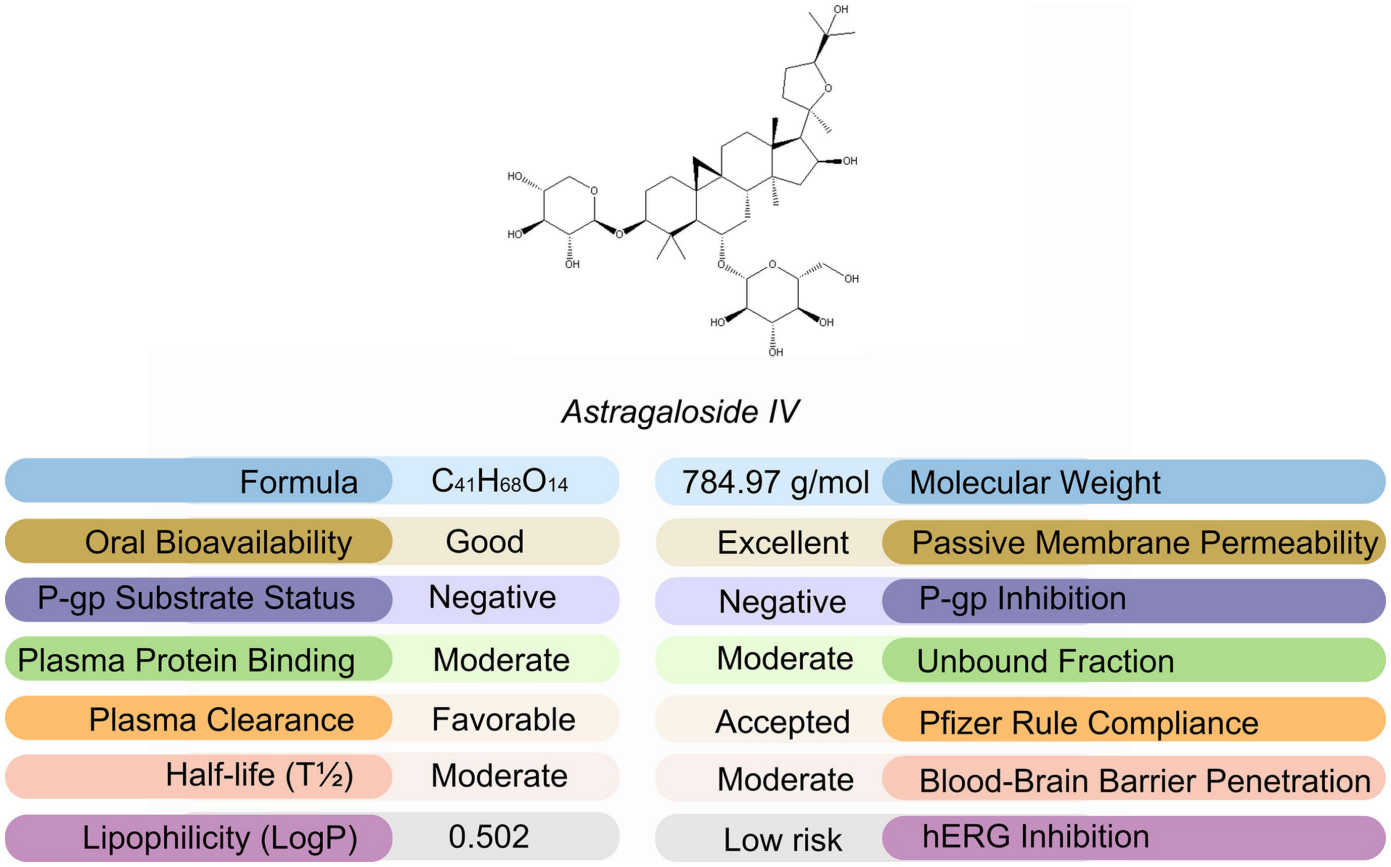
Molecular structure and key physicochemical properties of Astragaloside IV.

Astragalus membranaceus, a fundamental herb in Traditional Chinese Medicine, contains Astragaloside IV (AS-IV) as one of its principal bioactive compounds (Figure 1) (13). AS-IV has demonstrated a broad spectrum of pharmacological activities, including immunomodulatory (14), antioxidant (15), anti-aging (16), and cardio-renal protective effects (17). Notably, preliminary studies in recent years have suggested that AS-IV may exert a bone-protective potential by promoting osteoblast activity (18), inhibiting osteoclast formation or function (19), and attenuating oxidative stress damage to bone tissue (20). Despite growing interest, no study has yet delivered a comprehensive in-vivo assessment of AS-IV in a validated PMOP model that unifies bone-turnover markers, microarchitecture, histomorphometry, transcriptomic profiling and immunofluorescence validation. It also remains unresolved whether its actions involve osteoimmune control and Wnt signaling, both pivotal to PMOP pathogenesis.

Guided by this rationale, we hypothesized that AS-IV, a plant-derived bioactive compound with antioxidant and immunomodulatory properties, can mitigate ovariectomy (OVX)-induced osteoporosis. We proposed that AS-IV achieves this by rebalancing bone remodeling, alleviating oxidative stress, and modulating osteoimmune–Wnt signaling pathways. To test this hypothesis, we employed a standardized OVX Sprague–Dawley rat model and a multi-disciplinary approach, integrating systemic phenotyping, tissue-level analysis, and mechanistic pathway identification. This included RNA sequencing, targeted molecular docking, and multiplex immunofluorescence to validate key pathways implicated in bone metabolism.

## 2 Materials and methods

### 2.1 Experimental animals and ethics

Thirty 8-week-old healthy female Sprague–Dawley rats weighing 190–220 g were purchased from SPF (Beijing) Biotechnology Co., Ltd. [License No.: SCXK (Jing) 2024–0001]. All rats were acclimatized for 1 week in an SPF-grade environment (constant temperature and humidity, 12/12 h light/dark cycle). The experimental protocol was approved by the Biomedical Research Ethics Committee of Shanxi Datong University (Approval No.: 2024 M0021).

### 2.2 Drugs and reagents

Astragaloside IV (AS-IV, Meilunbio, MB1955); Nilestriol (Shanghai Haohong Biomedical Technology Co., Ltd., LY-ZJS100239); Sodium carboxymethyl cellulose (CMC-Na, Dalian Meilun Biotechnology Co., Ltd., MB1717); Paraformaldehyde (VICMED, VIH100); EDTA decalcification solution (Shanghai Siding Biotechnology Co., Ltd., SD0113); Rat Osteocalcin (OCN) ELISA Kit (Shanghai Xuanzekang Biotechnology Co., Ltd., XZK-6112); Rat C-terminal telopeptide of type I collagen (CTX-I) ELISA Kit (Shanghai Xuanzekang Biotechnology Co., Ltd., XZK-5343); Rat Parathyroid Hormone (PTH) ELISA Kit (Shanghai Xuanzekang Biotechnology Co., Ltd., XZK-6309); Rat Superoxide Dismutase (SOD) ELISA Kit (Shanghai Xuanzekang Biotechnology Co., Ltd., XZK-5781); Rat Malondialdehyde (MDA) ELISA Kit (Shanghai Xuanzekang Biotechnology Co., Ltd., XZK-5664); Hematoxylin and Eosin (H&E) Staining Kit (Shanghai Siding Biotechnology Co., Ltd., SD0146); Tyramide Signal Amplification (TSA) Multiplex Immunofluorescence Staining Kit (for 3 markers/4 colors).

### 2.3 Ovariectomy (OVX) model establishment and grouping

Rats were randomly divided into a sham-operated group (*n* = 5) and a modeling group (*n* = 25). Rats in the modeling group were anesthetized by intraperitoneal injection of 3% sodium pentobarbital (1 mL/kg) and underwent bilateral ovariectomy to replicate the postmenopausal osteoporosis model. The sham-operated group only had their ovaries exposed and an equivalent volume of fat tissue removed, without ovarian excision. Postoperatively, penicillin (40,000 U/kg) was administered intramuscularly for 3 consecutive days to prevent infection, and the animals’ status was monitored daily.

Six weeks post-surgery, the modeling group rats were randomly divided into 5 groups (n = 5/group): Model group, Positive control group (1.5 mg/kg Nilestriol), and AS-IV low, medium, and high dose groups (20/40/80 mg/kg).

The AS-IV doses (20, 40, and 80 mg/kg) were selected based on prior *in vivo* studies demonstrating biological activity within this range. Notably, a recent OVX mouse study reported bone-protective effects with AS-IV at 80 mg/kg (20), supporting the biological activity of doses within the range used in our study. Similar dose-escalation schemes have also been used in inflammatory models (21, 22). Accordingly, the three-dose design was adopted to evaluate potential dose responsiveness based on these published reports.

### 2.4 Drug administration

The reagents were prepared as follows: a solution of 400 mg astragaloside IV in 100 mL of 1% sodium carboxymethyl cellulose (CMC-Na).

Rats in each group received the corresponding drug by oral gavage once daily for 8 consecutive weeks. Sham and OVX model animals received vehicle by oral gavage (1% sodium carboxymethyl cellulose, CMC-Na) at a volume equivalent to the treatment groups. Body weight was measured weekly, and the administered dose was adjusted accordingly.

### 2.5 Sample collection

After the final administration of the treatments, rats were anesthetized using 3% sodium pentobarbital (1 mL/kg) via intraperitoneal injection. Once the animals were fully anesthetized, blood was collected from the abdominal aorta. Blood samples were centrifuged at 3000 rpm for 10 min to separate serum, which was stored at −80 °C for subsequent analysis. Following blood collection, the rats were euthanized by intraperitoneal injection of a lethal dose of 3% sodium pentobarbital (2 mL/kg) to ensure humane euthanasia. Bilateral femurs and the third lumbar vertebra (L3) were carefully dissected. One femur and the L3 vertebra were fixed in 4% paraformaldehyde for Micro-CT scanning and histological analysis. The contralateral femur was frozen at −80 °C for subsequent molecular biology detection.

### 2.6 Serum biomarker detection

Serum levels of bone metabolism markers (OCN, CTX-I, PTH) and oxidative stress indicators (SOD, MDA) were measured using ELISA kits according to the manufacturer’s instructions.

### 2.7 Bone microarchitecture analysis by micro-CT

The fixed femurs were scanned using a Micro-CT system (Pingsheng Medical, NMC-200). Scanning parameters were set as follows: voltage 80 kV, current 0.06 mA, exposure time 20 ms, and scan resolution 35 μm. Three-dimensional reconstruction was performed using the instrument’s software, and bone mineral density (BMD), bone volume fraction (BV/TV), trabecular number (Tb.N), and trabecular separation (Tb.Sp) were quantitatively analyzed.

### 2.8 Bone histomorphometric analysis (H&E staining)

Fixed femurs and vertebrae were decalcified in EDTA decalcification solution for 4 weeks, routinely embedded in paraffin, and sectioned into 5 μm thick slices. The sections were stained with Hematoxylin and Eosin (H&E). The morphology, number, thickness of trabecular bone, and changes in the bone marrow cavity were observed and compared under a light microscope.

### 2.9 mRNA-seq

Total RNA was extracted from three independent biological replicates per group. Sequencing libraries were constructed using oligo(dT) beads to enrich eukaryotic mRNA, followed by fragmentation and reverse transcription with random hexamer primers. Double-stranded cDNA was synthesized, end-repaired, adenylated, and ligated with Illumina adapters. Fragment size selection was performed, and the resulting libraries were amplified by PCR. Library quality was validated using the Agilent 2,100 Bioanalyzer. Sequencing was carried out on the Illumina HiSeq X-ten platform by Shanghai Oebiotech Medical Technology Co., Ltd. (Shanghai, China).

Raw sequencing reads were quality-controlled using FASTQC and preprocessed with NGS QC Toolkit v2.3.3 to obtain clean reads. Clean reads were aligned to the reference genome using Tophat/Bowtie2. Gene expression levels were quantified using FPKM with Bowtie2 and eXpress. Differential expression analysis was performed with the DESeq package using a negative binomial test. Significantly differentially expressed genes (DEGs) were identified with a *p*-value < 0.05 and |log2FC| > 0.58. Functional enrichment analysis of DEGs was conducted through Gene Ontology (GO) and KEGG pathway analyses using hypergeometric tests.

### 2.10 AS-IV high-dose treatment reverses OVX-induced transcriptomic alterations

To identify genes whose dysregulation induced by OVX was effectively reversed by AS-IV treatment, we performed a comparative analysis. Specifically, we first isolated genes that were up-regulated in OVX versus Sham and subsequently down-regulated in OVX-AS-IV-High versus OVX. Conversely, we identified genes that were down-regulated in OVX versus Sham and subsequently up-regulated in OVX-AS-IV-High versus OVX. The final set of rescued DEGs was defined as the union of these two antagonistic gene sets, representing transcripts that were significantly normalized by the high-dose AS-IV treatment.

### 2.11 Protein–protein interaction (PPI) network construction

The union of 36 rescued DEGs was subjected to PPI network analysis using the STRING database^1^ (version 12.0). Of the 36 input genes, 32 were successfully mapped to known protein interactors and finally obtained 7 interconnected hub proteins. The resulting PPI network was then visualized in Cytoscape (version 3.9.1).

### 2.12 Weighted gene co-expression network analysis (WGCNA)

To identify biologically significant co-expressed gene modules and explore the association between gene networks and the experimental phenotypes (Sham, OVX, OVX-AS-IV-High), we performed a weighted gene co-expression network analysis (WGCNA) using the WGCNA package (v1.73) in R (v4.0.3). Raw expression matrices were preprocessed by removing outliers via sample hierarchical clustering and filtering low-variance genes (retaining the top 75% by median absolute deviation). A signed network was constructed with a soft-thresholding power (*β*) selected via the scale-free topology criterion (*R*^2^ > 0.85). Dynamic tree-cutting (deepSplit = 2, minModuleSize = 30) was employed to identify co-expression modules, each of which was color-coded for visualization. Module–trait relationships were quantified by correlating module eigengenes (first principal component) with the clinical phenotypes using Pearson’s correlation (*p* < 0.05). A heatmap was generated to visualize module–phenotype associations with hierarchical clustering (Euclidean distance and complete linkage).

### 2.13 Functional enrichment analysis

To elucidate the biological functions and signaling pathways associated with the 36 core rescued targets, functional enrichment analysis was performed. Gene Ontology (GO) and Kyoto Encyclopedia of Genes and Genomes (KEGG) pathway analyses were conducted using the online bioinformatics platform Microbiome Bioinformatics (microbiomeX^2^). The entire set of genes quantified in the mRNA-seq dataset was used as the background reference. Enrichment was assessed using a hypergeometric test, and terms with a *p*-value of less than 0.05 were considered statistically significant.

### 2.14 Molecular docking analysis

To validate the binding potential between the core targets and the active compound Astragaloside IV (AS-IV), molecular docking was performed. The two-dimensional structure of AS-IV (CAS: 84687–43-4) was obtained from the PubChem database^3^ and prepared for docking by energy minimization and assignment of Gasteiger charges using AutoDock Tools.

The crystal structures of the target proteins were retrieved from the PDB database^4^. Specifically, the structures for Lep (PDB ID: 1AX8), Gfap (6A9P), Ptgs2 (5F19), Il22ra2 (3G9V), and GZMB (1FQ3) were downloaded. For proteins whose experimental structures were unavailable (Igfbp2, Wnt1, and Wnt10a), high-confidence predicted models from the AlphaFold database^5^ (AF-Q5JXC2-F1, AF-P04628-F1, and AF-Q9GZT5-F1, respectively) were employed.

All protein structures were prepared by removing water molecules and co-crystallized ligands, adding polar hydrogens, and assigning Kollman united atom charges. Docking simulations were carried out using AutoDock Vina software. The grid box was centered on the known active site of each protein, with dimensions large enough to allow the ligand free rotation. The docking pose with the most favorable (lowest) binding free energy (kcal/mol) was selected for each complex.

### 2.15 Immunofluorescence staining of bone tissue

The decalcified femora were then embedded in paraffin, and 4–5 μm thick sections were cut using a Cryostat (Leica Microsystems Shanghai Co., Ltd., CM1950). These sections were deparaffinized in xylene I (15 min) and xylene II (15 min), rehydrated through graded ethanol (absolute ethanol I, 5 min; absolute ethanol II, 5 min; 95% ethanol, 5 min; 85% ethanol, 5 min; 75% ethanol, 5 min), and rinsed in distilled water. Antigen retrieval was carried out in alkaline EDTA buffer (pH 9.0) using microwave heating, followed by natural cooling and washing in PBS (pH 7.4) on a rocker (3 × 5 min). Endogenous peroxidase activity was quenched with 3% hydrogen peroxide for 15 min at room temperature in the dark, followed by PBS washes (3 × 5 min). After outlining the tissue area with a hydrophobic barrier pen, sections were blocked with 3% BSA in PBS for 30 min at room temperature. Primary antibodies diluted in antibody diluent were applied and incubated at 4 °C overnight; the primary antibodies were as follows: PTGS2 (ABclonal, A3560, rabbit, 1:200), IGFBP2 (Proteintech, 66,644-1-IG, mouse, 1:200), WNT1 (ABclonal, A2475, rabbit, 1:200), LEP (bioss, bs-0409R, rabbit, 1:200), GFAP (Proteintech, 60,190-1-IG, mouse, 1:1000), *β*-catenin (huilanbio, ABB3523, rabbit, 1:200), and NF-κB p65 (Cell Signaling Technology, 8,242, rabbit, 1:200), with EDTA-based retrieval used for all targets. After PBS washing (3 × 5 min), sections were incubated with the corresponding HRP-conjugated secondary antibody for 50 min at room temperature in the dark, washed again in PBS (3 × 5 min), and then reacted with the ready-to-use fluorophore reaction solution for 2–15 min at room temperature followed by PBS washes (3 × 5 min). For sequential multi-target labeling, antibody stripping was performed using a dedicated mIHC stripping buffer prewarmed to 37 °C (5–20 min, repeated twice), after which the staining cycle (blocking/primary/HRP-secondary/tyramide reaction) was repeated with an alternative tyramide fluorophore. Nuclei were counterstained with ready-to-use DAPI for 5–20 min at room temperature in the dark, and sections were mounted with an anti-fade mounting medium. Fluorescence signals were visualized and recorded using a fluorescence microscope/confocal imaging system as available, including a Nikon Eclipse CI-S microscope equipped with a Nikon DS-U3 imaging system, and/or whole-slide scanning with a Pannoramic MIDI scanner (3DHISTECH).

### 2.16 Statistical analysis

Data are expressed as mean ± SD. Statistical analyses and plotting were performed in GraphPad Prism 10. Group differences were assessed using one-way ANOVA followed by Dunnett’s multiple comparisons test versus the designated control group. *p* < 0.05 was considered statistically significant.

## 3 Results

### 3.1 AS-IV reverses bone metabolic disorders in OVX rat

**Figure 2:**
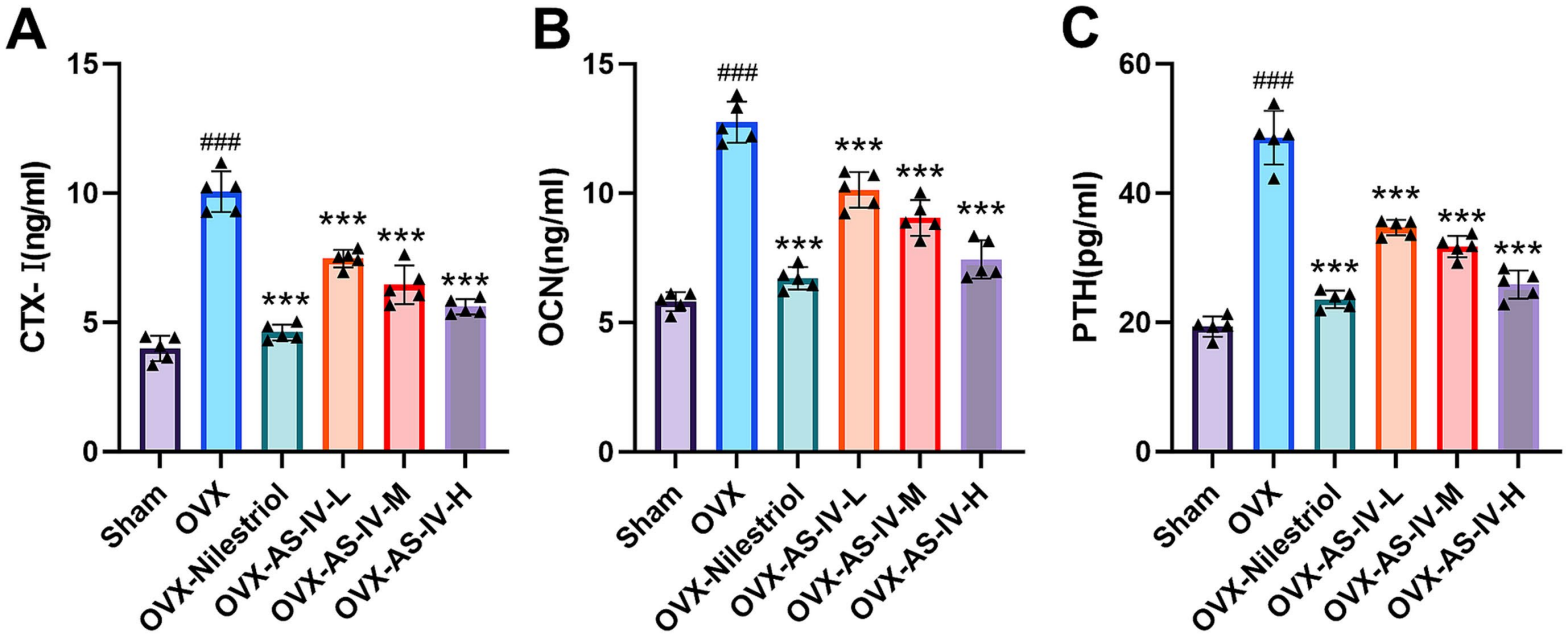
Effects of Astragaloside IV on serum CTX-I, OCN, and PTH in osteoporotic rats. **(A)** C-terminal telopeptide of type I collagen (CTX-I, ng/ml); **(B)** osteocalcin (OCN, ng/ml); **(C)** parathyroid hormone (PTH, pg./ml). Statistical significance: ^###^*p* < 0.001 vs. sham group, ***p < 0.001 vs. OVX group.

To assess AS-IV’s systemic effects on bone metabolism, we quantified key serum biomarkers. As shown in Figure 2, the OVX rats had significantly higher OCN, CTX-I, and PTH than Sham (all *p* < 0.001), confirming a high–bone-turnover state.

Treatment with AS-IV effectively mitigated these metabolic disturbances. Regarding bone resorption, AS-IV administration significantly reduced the elevated CTX-I levels in a dose-dependent manner, with the high-dose group showing the most pronounced effect (Figure 2A). For bone formation, the OVX-AS-IV-H also significantly lowered OCN levels, demonstrating comparable efficacy to the positive control drug (Figure 2B). Furthermore, all AS-IV treatment groups significantly suppressed PTH levels (Figure 2C). In summary, our results demonstrate that AS-IV re-establishes bone metabolic equilibrium in OVX rats by concurrently stimulating bone formation and suppressing bone resorption.

### 3.2 AS-IV alleviates oxidative stress levels in OVX rats

**Figure 3:**
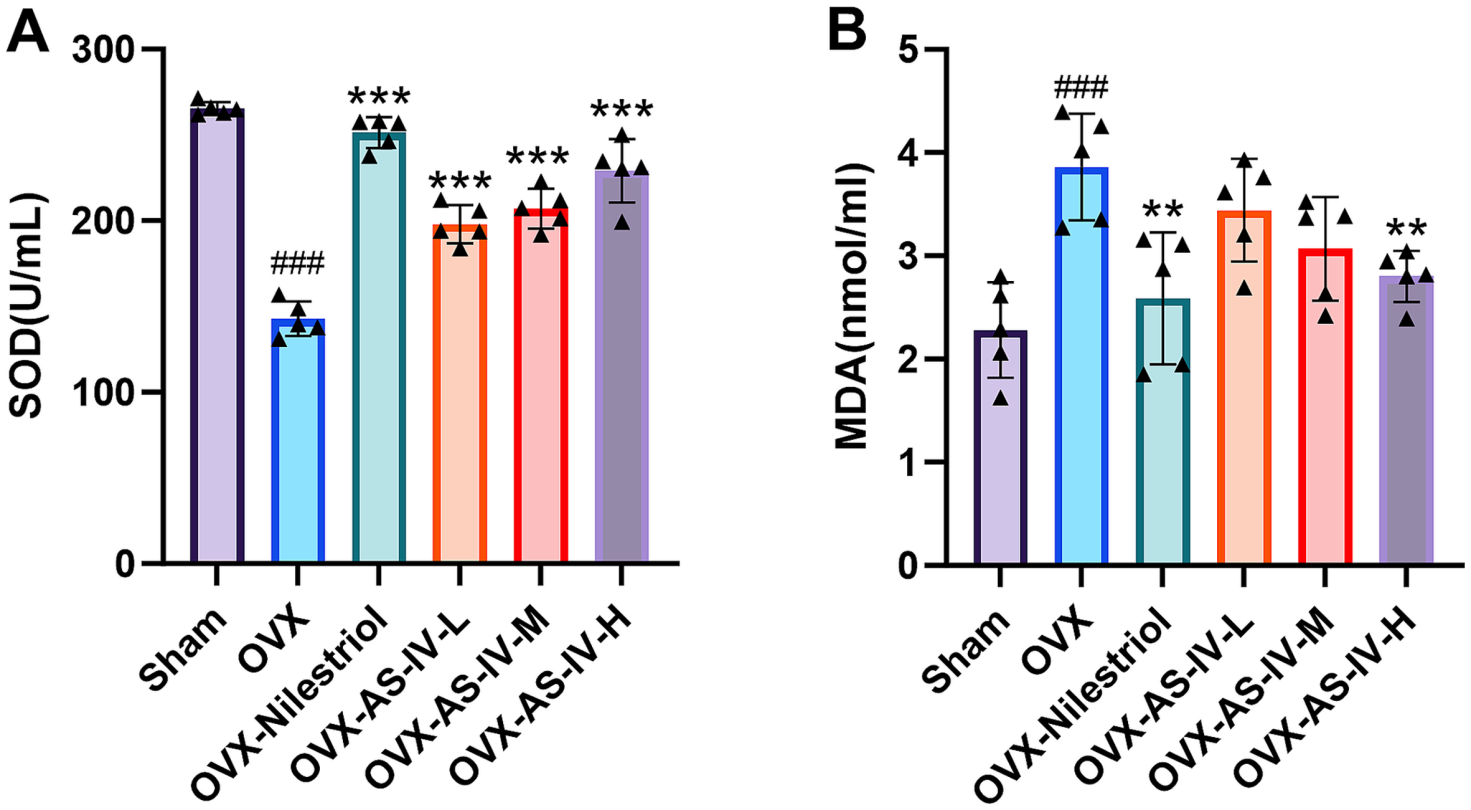
Effects of Astragaloside IV on oxidative stress markers in osteoporotic rats. **(A)** Superoxide dismutase (SOD, U/mL); **(B)** malondialdehyde (MDA, nmol/ml). Statistical significance: ^###^*p* < 0.001 vs. sham group, ^***^p < 0.001 vs. ovx group, **p < 0.01 vs. group.

To further characterize AS-IV’s antioxidant activity, we first confirmed that ovariectomy produced a robust pro-oxidant shift: the OVX group showed a significant reduction in serum SOD activity together with a concomitant rise in MDA content (both *p* < 0.001; Figure 3). Administering AS-IV reversed this pattern in a graded fashion. Across doses, SOD activity was restored (*p* < 0.001), and at the highest dose MDA levels were simultaneously suppressed (*p* < 0.01), indicating coordinated improvement of enzymatic defense and lipid peroxidation status. The extent of change in the high-dose AS-IV cohort closely matched that achieved by the positive control. Taken together, these observations substantiate the antioxidant capacity of AS-IV and support attenuation of oxidative stress as a plausible mechanistic contributor to its bone-protective effects in the OVX setting.

### 3.3 AS-IV improves bone microarchitecture in OVX rats

**Figure 4:**
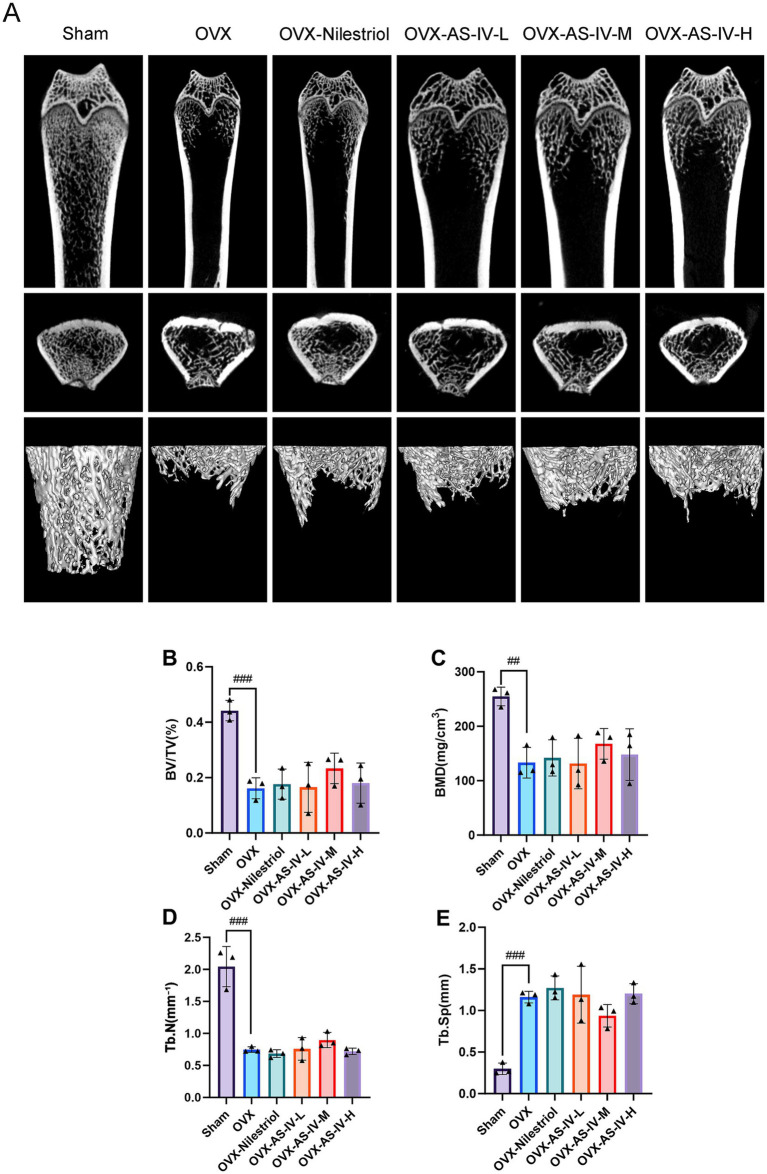
Rat femoral bone tissue micro-CT scan and 3D reconstruction. **(A)** Micro-CT images of the femur: 2D coronal, 2D cross-sectional, and 3D trabecular views; **(B)** bone volume/total volume (BV/TV, %); **(C)** bone mineral density (BMD, mg/cm^3^); **(D)** trabecular number (Tb.N, 1/mm); **(E)** trabecular separation (Tb.Sp, mm). ###*p* < 0.001 vs. sham group; ##*p* < 0.01 vs. sham group. No statistically significant differences were observed between AS-IV treatment groups and the OVX group (*p* > 0.05) for BMD, BV/TV, Tb.N, or Tb.Sp.

Micro-CT–based three-dimensional reconstructions of the femur revealed marked structural differences between Sham and OVX animals (Figure 4A). In the Sham group, trabeculae were numerous, evenly distributed, and interconnected, forming a compact and continuous cancellous network. In contrast, OVX rats exhibited classical osteoporotic features, including reduced trabecular number, discontinuity of trabecular connections, widened inter-trabecular spaces, and enlarged marrow cavities, indicating pronounced trabecular deterioration following ovariectomy.

Quantitative analyses further confirmed significant structural impairment in the OVX group compared with Sham controls (Figures 4B–E). Specifically, OVX rats showed significantly decreased BMD (*p* < 0.01), BV/TV (*p* < 0.001), and Tb.N (*p* < 0.001), accompanied by a significant increase in Tb.Sp (*p* < 0.001). These findings validate the successful establishment of the OVX-induced osteoporotic phenotype.

When compared with the OVX group, neither the positive control (Nilestriol) nor the AS-IV low-, medium-, or high-dose groups demonstrated statistically significant differences in BMD, BV/TV, Tb.N, or Tb.Sp (all *p* > 0.05). Although numerical shifts toward improved parameters were observed in treated groups, these changes did not reach statistical significance under the current experimental conditions. Several factors may contribute to this lack of significance, including the small sample size, inherent within-group variability, and suboptimal treatment duration, which may have limited the statistical power of the study. Accordingly, structural rescue of trabecular microarchitecture by AS-IV was not demonstrated in this study.

Micro-CT scans and 3D reconstruction images and data of rat vertebrae tissue are detailed in Supplementary materials S1.

### 3.4 Morphological structure of bone tissue in AS-IV-repaired OVX rats

**Figure 5:**
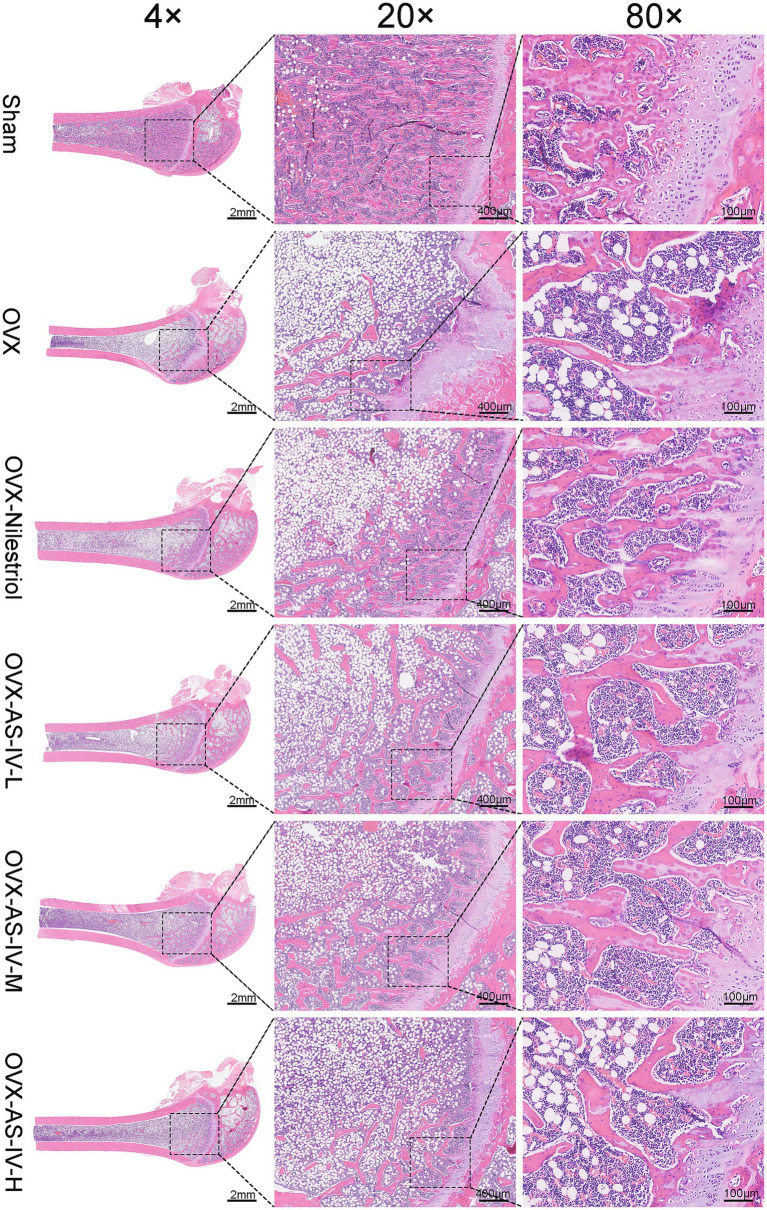
H&E histology of decalcified paraffin sections from rat femoral trabecular bone shown as a three-magnification composite (4×, 20×, 80×; left-to-right within each group). Scale bars as indicated.

Histological examination of H&E–stained femora corroborated the micro-CT findings (Figure 5). In Sham rats, trabeculae were thick, evenly distributed, and interconnected into a continuous lattice. By contrast, OVX sections exhibited the classical osteoporotic phenotype—thinned and fragmented trabeculae, widened inter-trabecular spaces, and conspicuously enlarged marrow cavities. Notably, AS-IV–treated groups showed qualitative restoration of architecture: trabeculae appeared thicker with improved continuity and fewer perforations, and marrow cavities were reduced. Taken together, these histological features are consistent with the imaging readouts and support a protective effect of AS-IV on trabecular microarchitecture. H&E histology of decalcified paraffin sections from rat vertebral trabecular bone shown as a three-magnification composite (4×, 20×, 80×; left-to-right within each group) are detailed in Supplementary materials S2.

### 3.5 mRNA-seq

To delineate the transcriptomic alterations underlying the OVX-induced condition and the therapeutic effect of AS-IV, we performed mRNA sequencing on bone tissue from the Sham, OVX, and OVX-AS-IV-High groups. Differential expressed genes (DEGs) analysis was conducted with thresholds set at a *p*-value < 0.05 and an absolute log2 fold-change (|log2FC|) > 1.

**Figure 6:**
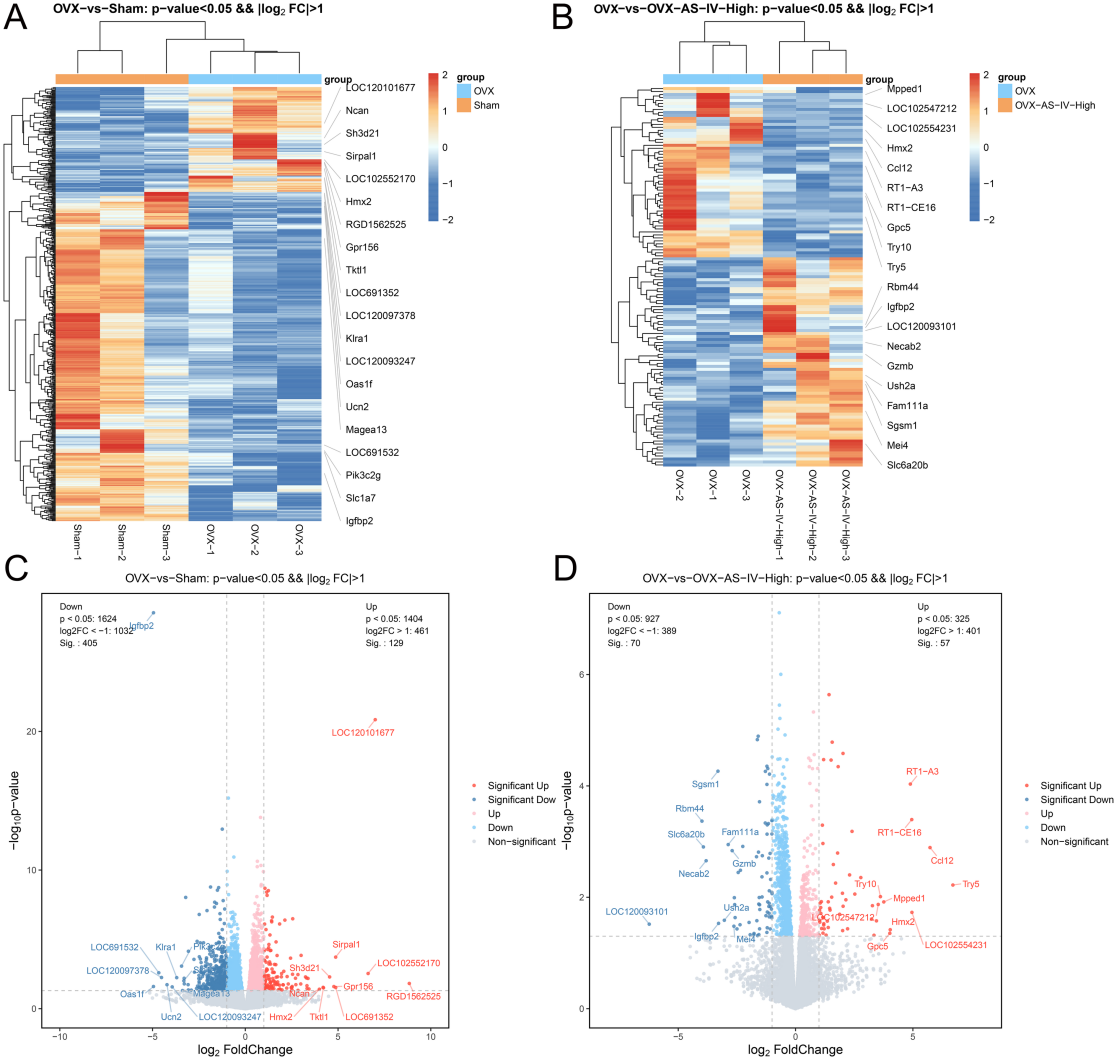
Transcriptomic profiling of OVX-induced changes and AS-IV intervention. **(A)** Hierarchical clustering heatmap of DEGs from OVX vs. Sham comparison, showing clear separation between the two groups; **(B)** Heatmap of DEGs from OVX vs. OVX-AS-IV-High comparison, illustrating the modulatory effect of AS-IV on the gene expression profile; **(C)** Volcano plot of DEGs in OVX vs. Sham. Significantly upregulated genes (red) and downregulated genes (blue) are highlighted (*p*-value < 0.05, |log2FC| > 1). Key DEGs are labeled; **(D)** Volcano plot of DEGs in OVX vs. OVX-AS-IV-High, highlighting genes significantly altered by AS-IV treatment.

Comparative analysis between the OVX and Sham groups identified substantial transcriptomic dysregulation. A total of 16,348 genes were significantly dysregulated, with 534 genes meeting the more stringent significance criteria (*p* < 0.05 & |log2FC| > 1). This included 129 genes significantly up-regulated and 405 genes significantly down-regulated in the OVX group compared to Sham (Figure 6C, Volcano plot). The expression patterns of these DEGs are vividly displayed in a heatmap, which clearly segregates the OVX and Sham samples and highlights distinct gene clusters, including notably altered genes such as Ncan, LOC120101677, and Igfbp2 (Figure 6A).

Subsequent comparison between the OVX and OVX-AS-IV-High groups aimed to identify genes modulated by the AS-IV treatment. This analysis revealed that AS-IV intervention induced a significant transcriptional response, leading to the reversal of a subset of OVX-induced changes. We identified 127 genes that were significantly dysregulated following AS-IV treatment (*p* < 0.05 and |log2FC| > 1), with 57 genes up-regulated and 70 genes down-regulated (Figure 6D, Volcano plot). The corresponding heatmap demonstrates a clear normalization effect of AS-IV on the global gene expression profile, as seen by the distinct clustering of the treatment group away from the OVX group (Figure 6B).

Taken together, these extensive transcriptomic profiles not only validate the widespread gene expression changes triggered by OVX but also highlight the strong therapeutic potential of high-dose AS-IV, which normalizes a significant proportion of these alterations. This, in turn, provides a molecular foundation for understanding its bone-protective effects.

### 3.6 AS-IV high-dose treatment reverses OVX-induced transcriptomic alterations

**Figure 7:**
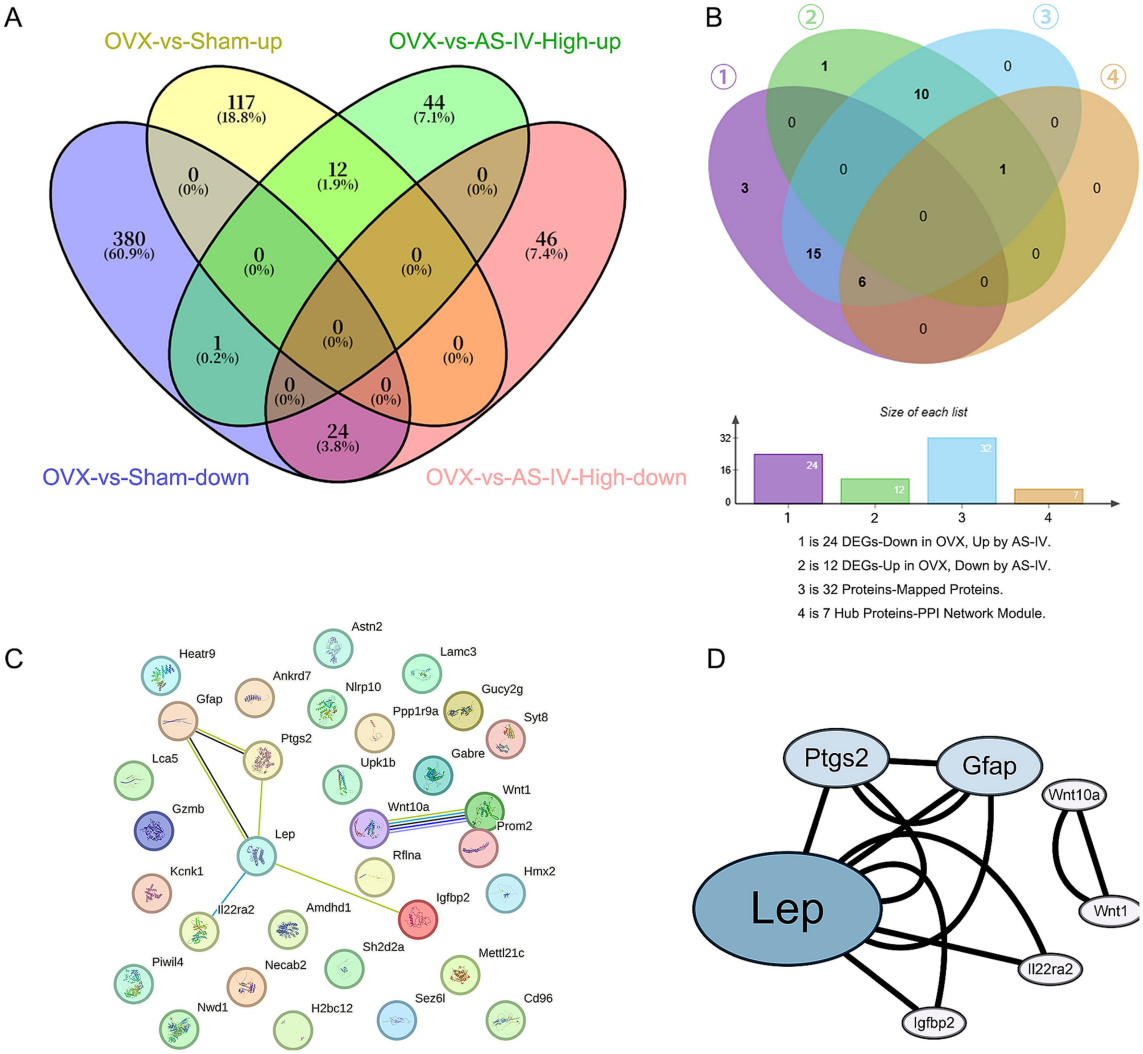
Identification and protein interaction network of AS-IV-rescued DEGs. **(A)** Venn diagrams illustrate the identification of 36 “AS-IV-rescued” DEGs: 24 that were down-regulated by OVX and up-regulated by AS-IV, and 12 that were up-regulated by OVX and down-regulated by AS-IV. **(B)** Venn diagrams illustrate the bioinformatics filtering process. **(C)** The 32 mapped protein candidates encoded by the rescued DEGs, which were retrieved from the STRING database for PPI network construction. **(D)** The PPI network module comprising 7 interconnected hub proteins. The node size represents the degree of each protein, indicating its connectivity within the network. Larger nodes correspond to proteins with a higher number of interactions, suggesting their potential central role in the network.

By intersecting the opposingly regulated genes from these two comparisons, we identified a core set of 36 genes that were dysregulated by OVX and subsequently reversed by AS-IV treatment. The mRNA-seq analysis identified 24 genes that were down-regulated in OVX versus Sham but up-regulated after AS-IV treatment, and 12 genes that exhibited the opposite pattern (up-regulated in OVX and down-regulated by AS-IV). The union of these 36 rescued genes was subjected to protein–protein interaction (PPI) network analysis. Of these, 32 gene products were mapped onto the PPI network, the visualization of which is presented in Figure 7A. We constructed Venn diagrams in Figure 7B to illustrate the bioinformatics filtering process. ① represents 24 genes that were down-regulated in the OVX group (vs. Sham) and up-regulated by AS-IV treatment (vs. OVX). ② represents 12 genes that were up-regulated in OVX and down-regulated by AS-IV. The union of ① and ② defines the 36 “AS-IV-rescued” DEGs. ③ denotes the 32 proteins successfully mapped from the 36 rescued DEGs in the STRING database. ④ indicates the interconnected 7-protein module extracted from the PPI network.

### 3.7 PPI network analysis

To uncover the functional relationships and potential protein-level interactions among the 36 rescued DEGs, we constructed a protein–protein interaction (PPI) network using the STRING database in Figure 7C. Among the 36 input genes, 32 gene products had corresponding protein entries in the database and were included in the analysis. Visualization and subsequent analysis in Cytoscape revealed a connected network comprising only 7 proteins, including Lep, Ptgs2, Gfap, and Wnt10a. This highly interconnected module suggests their potential core functional role in the therapeutic mechanism of AS-IV, as depicted in Figure 7D.

### 3.8 Weighted gene co-expression network analysis identifies clinically relevant gene modules

To decipher the coordinated gene expression changes underlying the OVX-induced condition and the therapeutic effect of AS-IV, we constructed a weighted gene co-expression network from the transcriptomes of the Sham, OVX, and OVX-AS-IV-High groups.

**Figure 8:**
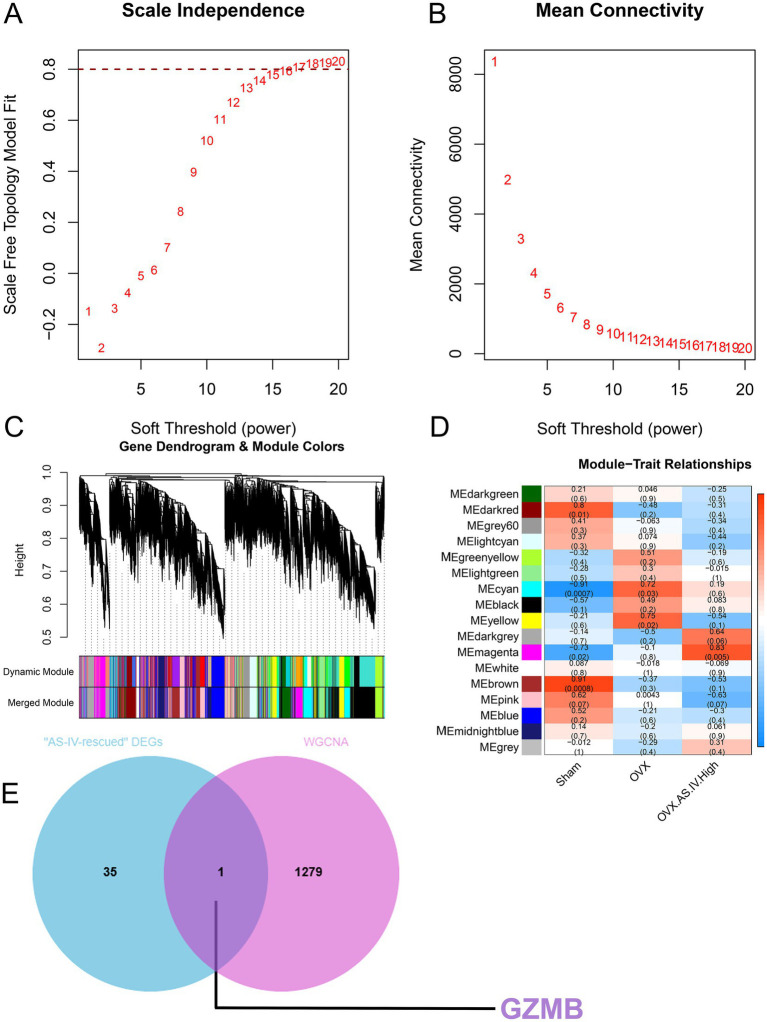
Weighted gene co-expression network analysis (WGCNA). **(A,B)** Optimization of the soft threshold for network construction; **(C)** Dendrogram illustrating the hierarchical clustering results of WGCNA; **(D)** Module–trait associations. The heatmap displays correlation coefficients between module eigengenes and experimental groups; **(E)** Identification of the hub gene GZMB through integration of rescued DEGs and WGCNA.

Initially, we assessed the network topology to determine an appropriate soft-thresholding power (*β*). The analysis of scale independence confirmed that a power of β = 16 was the lowest value that achieved a scale-free topology fit index (*R*^2^) of approximately 0.8, ensuring a biologically meaningful, scale-free network (Figure 8A). The corresponding analysis of mean connectivity demonstrated that the chosen power maintained a sufficiently high mean connectivity for robust module detection (Figure 8B).

Using this soft-thresholding power, we constructed a gene co-expression network and identified co-expression modules via dynamic tree cutting. This procedure yielded distinct, color-coded modules from the gene dendrogram (Figure 8C), each representing a group of genes with highly synchronized expression profiles across the samples.

Crucially, the module-trait relationship analysis revealed several modules with significant associations with the experimental phenotypes (Figure 8D). The midnightblue module exhibited a strong positive correlation with the OVX group (correlation = 0.91, *p* = 0.0008), suggesting that its expression profile is highly specific to the disease state. Conversely, the gray module showed a pronounced negative correlation with OVX (correlation = −0.91, *p* = 0.0007), indicating its potential role as a protective signature that is suppressed in the model. Furthermore, the yellow module demonstrated a significant positive correlation with the AS-IV treatment group (correlation = 0.75, *p* = 0.02), highlighting its potential involvement in the therapeutic mechanism of AS-IV. The magenta module was also notably correlated with the AS-IV treatment (correlation = 0.83, *p* = 0.005). WGCNA revealed a distinct module (magenta) of 1,280 genes whose expression pattern was highly and significantly correlated with the AS-IV intervention (*r* = 0.83, *p* = 0.005). This strong positive correlation indicates that the expression levels of these 1,280 genes increased in a coordinated manner specifically in response to AS-IV treatment. Consequently, this gene network represents a transcriptomic signature of the drug’s effect and was extracted for further investigation into the underlying molecular processes.

Considering their robust and statistically significant associations with the key experimental conditions, the midnightblue, gray, yellow, and magenta modules were deemed the most clinically relevant. These key modules were therefore prioritized for subsequent functional enrichment analysis to elucidate the biological processes and pathways they represent in the context of OVX and AS-IV treatment. A Venn diagram illustrates the intersection between the 36 AS-IV-rescued DEGs and the gene members of the key module (midnightblue module) identified by WGCNA, which was strongly and positively correlated with the OVX phenotype. The overlap identifies Gzmb (Granzyme B) as a key candidate hub gene, linking the transcriptomic reversal by AS-IV to the disease-associated co-expression network (Figure 8E).

### 3.9 Functional enrichment analysis reveals key biological processes and pathways of the rescued targets

To elucidate the biological functions of the core targets rescued by AS-IV, we performed systematic Gene Ontology (GO) and Kyoto Encyclopedia of Genes and Genomes (KEGG) pathway enrichment analyses on two distinct gene sets: the full set of 36 rescued DEGs and the refined list of 8 hub genes (7 from the PPI network and 1 from WGCNA).

**Figure 9:**
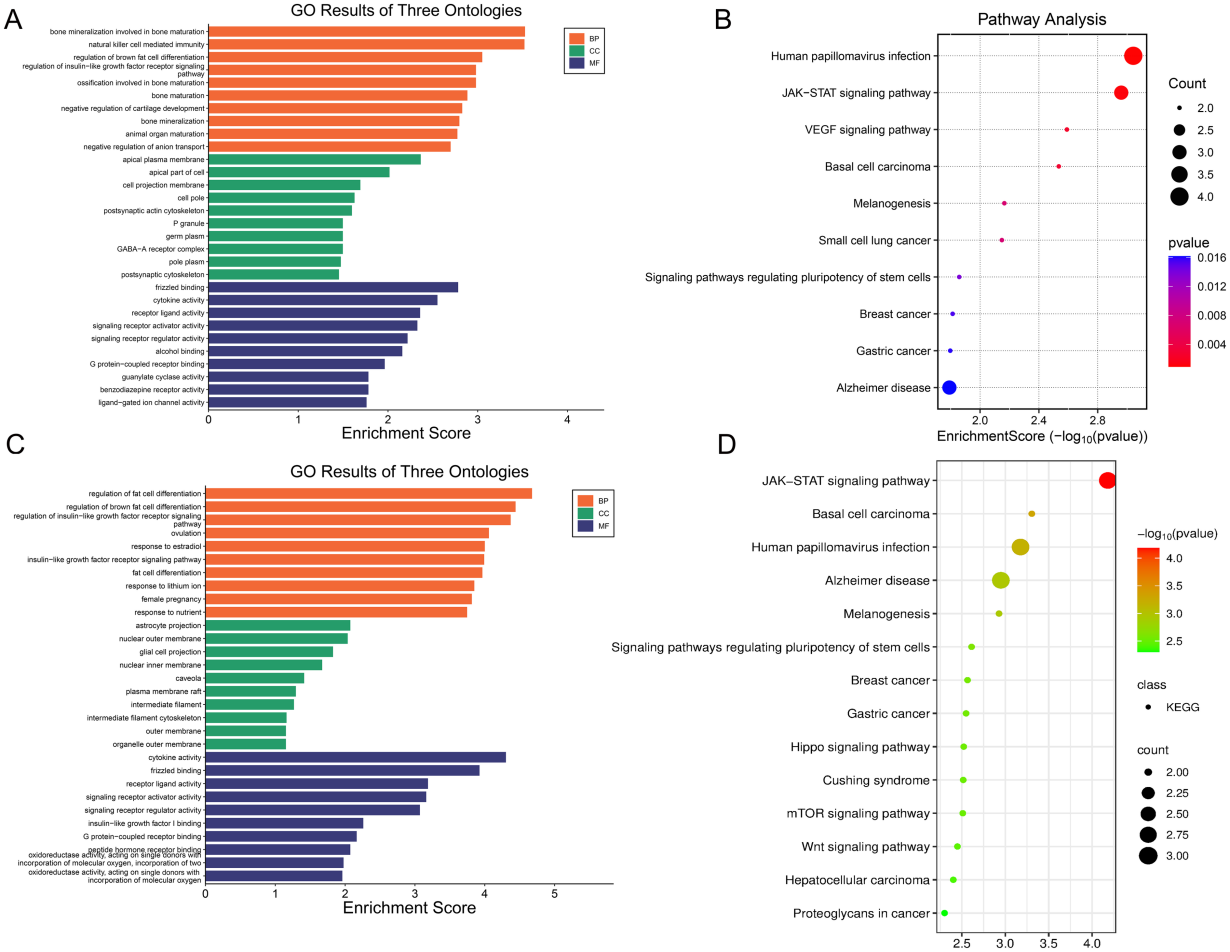
Functional enrichment analysis of the core targets rescued by Astragaloside IV (AS-IV). **(A)** Bar plot of significantly enriched Gene Ontology (GO) terms of the 36 core rescued targets, categorized by molecular function and biological process. The terms are associated with receptor signaling and neuronal development; **(B)** Bubble plot of significantly enriched KEGG pathways of the 36 core rescued targets. The size of the bubble represents the number of genes in the pathway (Count), and the color intensity corresponds to the statistical significance (−log10(*p*-value)). Key pathways such as the JAK–STAT and VEGF signaling pathways are highlighted; **(C)** GO enrichment analysis of the 8 hub genes (7 from the PPI network and 1 from WGCNA). The bar plot shows the top enriched functional terms associated with these key targets; **(D)** KEGG pathway enrichment analysis of the 8 hub genes. The bubble plot highlights the most significant pathways, including the Wnt signaling pathway, which is prominently enriched.

Functional analysis of the 36 core rescued targets demonstrated their significant involvement in specific biological processes. The GO analysis indicated a strong association with terms such as the regulation of fat cell differentiation and pathways related to insulin-like growth factor receptor signaling (Figure 9A). Concurrently, KEGG pathway enrichment highlighted that these 36 targets are significantly enriched in several critical signaling cascades and disease-related pathways. The most prominent among these were the JAK–STAT signaling pathway and the VEGF signaling pathway, alongside multiple cancer pathways such as Basal cell carcinoma, Small cell lung cancer, and Breast cancer (Figure 9B).

We subsequently focused on the 8 hub genes to pinpoint the most central mechanisms. Strikingly, the GO and KEGG analysis of this small yet critical gene set confirmed their convergence on several high-impact pathways (Figures 9C,D). The JAK–STAT pathway remained the top enrichment signal, underscoring its central importance in this context. In addition, Wnt signaling was explicitly identified, providing direct mechanistic support for the observed anabolic actions of AS-IV on bone formation.

Taken together, the enrichment results indicate that AS-IV acts therapeutically by tuning an integrated network of targets embedded across the JAK–STAT, VEGF, and Wnt signaling axes—pathways that occupy central positions in osteoimmunology and govern the balance of bone remodeling.

### 3.10 Molecular docking validates stable binding between AS-IV and core targets

**Figure 10:**
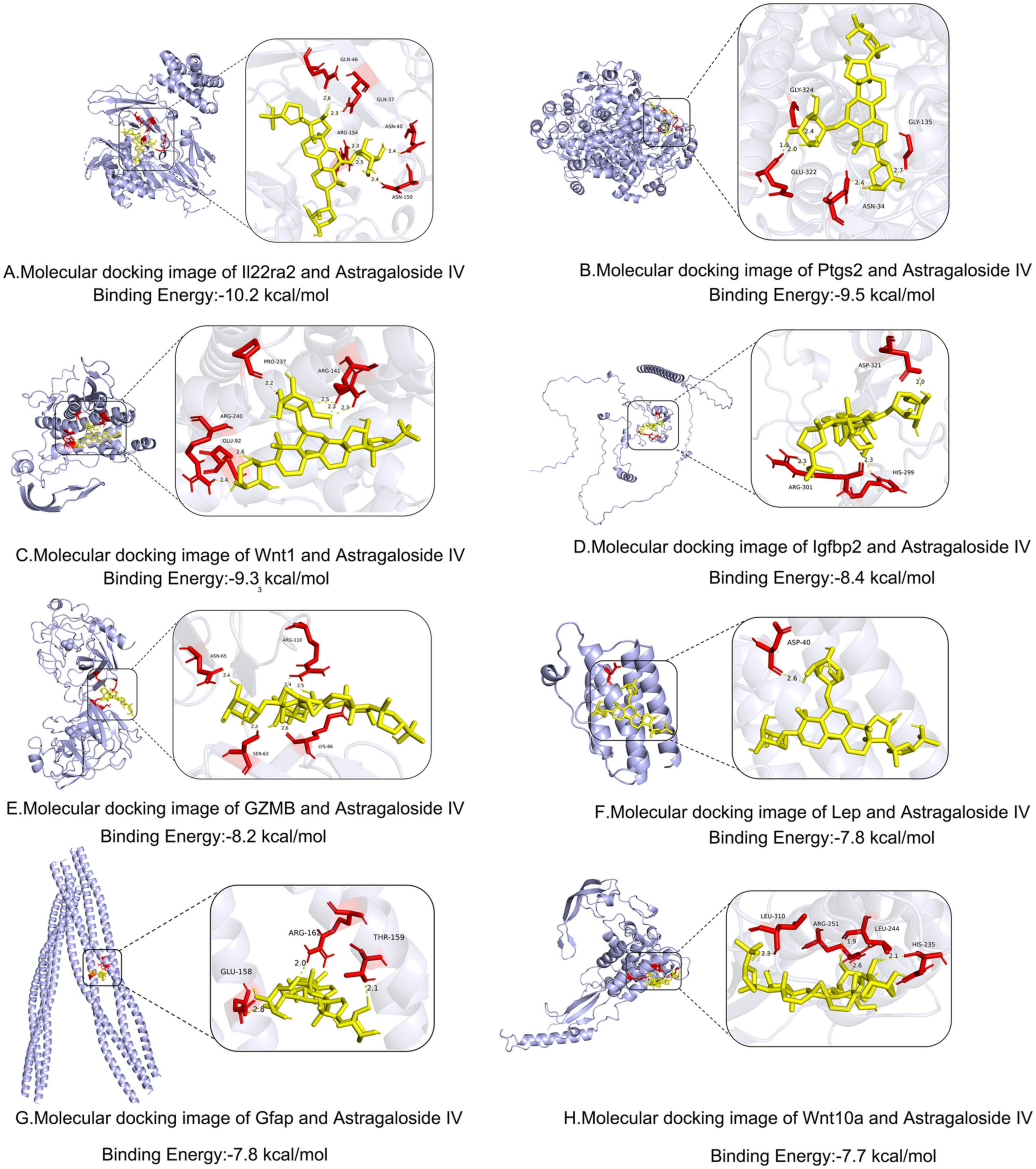
Molecular docking models of Astragaloside IV (AS-IV) binding to the core targets. Representative binding poses of AS-IV (shown in stick representation) within the predicted binding pockets of the key target proteins. The calculated binding free energy (kcal/mol) for each complex is indicated. **(A)** Il22ra2 (−10.2 kcal/mol), **(B)** Ptgs2 (−9.5 kcal/mol), **(C)** Wnt1 (−9.3 kcal/mol), **(D)** Igfbp2 (−8.4 kcal/mol), **(E)** GZMB (−8.2 kcal/mol), **(F)** Lep (−7.8 kcal/mol), **(G)** Gfap (−7.8 kcal/mol), **(H)** Wnt10a (−7.7 kcal/mol). These results demonstrate stable binding interactions between the therapeutic compound AS-IV and all eight core targets identified from the PPI network and WGCNA, with Il22ra2 and Ptgs2 showing the strongest binding affinity.

To substantiate the bioinformatics predictions, we investigated the direct interactions between the therapeutic compound AS-IV and the eight core targets (7 from the PPI network and 1 from WGCNA) using molecular docking. The results demonstrated that AS-IV could spontaneously bind to all eight targets with favorable binding free energies below −7.7 kcal/mol, indicating stable interactions (Table 1 and Figure 10).

**Table 1:** Molecular docking analysis result.

Protein	PDB ID	Molecule	CAS	Free Energy (kcal/mol)
Il22ra2	3G9V	Astragaloside IV	84,687-43-4	−10.2
Ptgs2	5F19	Astragaloside IV	84,687-43-4	−9.5
Wnt1	AF-P04628-F1	Astragaloside IV	84,687-43-4	−9.3
Igfbp2	AF-Q5JXC2-F1	Astragaloside IV	84,687-43-4	−8.4
GZMB	1FQ3	Astragaloside IV	84,687-43-4	−8.2
Lep	1AX8	Astragaloside IV	84,687-43-4	−7.8
Gfap	6A9P	Astragaloside IV	84,687-43-4	−7.8
Wnt10a	AF-Q9GZT5-F1	Astragaloside IV	84,687-43-4	−7.7

Notably, Il22ra2 exhibited the strongest binding affinity with AS-IV, yielding a free energy of −10.2 kcal/mol. Ptgs2 and Wnt1 also showed exceptionally high binding stability, with energies of −9.5 kcal/mol and −9.3 kcal/mol, respectively. The binding energies for the remaining targets, including Igfbp2 (−8.4 kcal/mol), GZMB (−8.2 kcal/mol), Lep (−7.8 kcal/mol), Gfap (−7.8 kcal/mol), and Wnt10a (−7.7 kcal/mol), were all significantly negative, confirming robust binding capabilities.

These *in silico* findings provide strong computational evidence that AS-IV can directly interact with the key network targets, particularly Il22ra2 and Ptgs2, thereby offering a structural basis for its observed pharmacological effects.

### 3.11 Immunofluorescence validation

**Figure 11:**
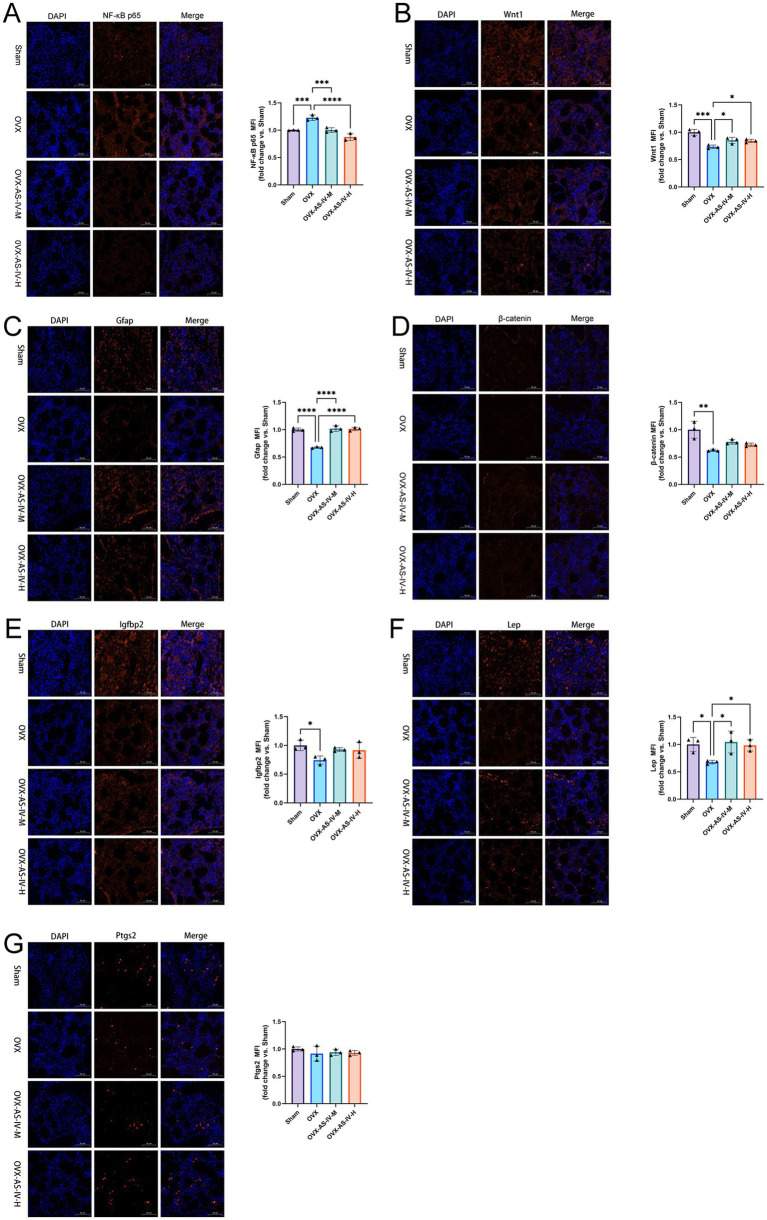
Multiplex immunofluorescence imaging and semi-quantification of target proteins in femoral trabecular bone. Representative multiplex immunofluorescence images showing DAPI (nuclei), target protein staining, and merged overlays in the femoral trabecular region from Sham, OVX, and OVX-AS-IV-M treated rats. Panels show staining for **(A)** NF-κB p65, **(B)** Wnt1, **(C)** Gfap, **(D)** β-catenin, **(E)** Igfbp2, **(F)** Lep, and **(G)** Ptgs2. Scale bar, 50 μm. The corresponding semi-quantification of mean fluorescence intensity (MFI) for each marker is presented to the right of each image panel, normalized to the Sham group (set to 1). Data are presented as mean ± SD with individual data points overlaid. Statistical comparisons were performed using one-way ANOVA followed by Dunnett’s multiple-comparisons test. *p < 0.05, **p < 0.01, ***p < 0.001.

To determine whether transcriptome-enriched pathways were reflected at the protein level, multiplex immunofluorescence was performed in the femoral trabecular region, and mean fluorescence intensity (MFI) was quantified for Lep, Ptgs2, Gfap, Igfbp2, Wnt1, *β*-catenin, and NF-κB p65 (Figures 11A–G).

Compared with the Sham group, OVX rats exhibited significantly increased NF-κB p65 immunoreactivity (*p* < 0.001), indicating activation of inflammatory signaling in trabecular bone. In parallel, Wnt1 expression was significantly reduced in OVX animals (*p* < 0.001 vs. Sham), and β-catenin levels were also decreased (*p* < 0.05). Gfap expression was markedly lower in OVX rats compared with Sham controls (*p* < 0.0001). Similarly, Lep and Igfbp2 fluorescence intensities were reduced in OVX bone relative to Sham (both *p* < 0.05). In contrast, Ptgs2 protein abundance did not differ significantly among groups.

Following AS-IV administration, NF-κB p65 expression was significantly reduced in both the mid-dose (*p* < 0.001) and high-dose groups (*p* < 0.0001) compared with OVX, indicating dose-associated attenuation of OVX-induced inflammatory activation. Gfap expression was significantly increased in both mid- and high-dose groups relative to OVX (both *p* < 0.0001). Wnt1 fluorescence intensity was also elevated in AS-IV–treated animals compared with OVX (mid-dose and high-dose, both *p* < 0.05), although the magnitude of restoration remained partial.

In contrast, β-catenin did not show significant differences between OVX and either AS-IV–treated group despite its reduction relative to Sham. Igfbp2 expression, although decreased in OVX compared with Sham, did not exhibit statistically significant recovery following AS-IV treatment (both *p* > 0.05 vs. OVX). Lep fluorescence intensity was significantly higher in both AS-IV–treated groups compared with OVX (both *p* < 0.05), indicating responsiveness at the protein level. Ptgs2 immunoreactivity remained unchanged across all groups.

Collectively, these findings demonstrate robust suppression of OVX-induced NF-κB activation by AS-IV and modulation of selected osteoimmune- and Wnt-related components, particularly Gfap and Wnt1. However, not all transcript-identified candidates exhibited concordant protein-level restoration, underscoring partial alignment between transcriptional enrichment and local protein expression within the trabecular microenvironment.

## 4 Discussion

This study utilized the ovariectomized (OVX) rat, an established model of postmenopausal osteoporosis (PMOP), to evaluate Astragaloside IV (AS-IV) across graded doses. Our results demonstrate that AS-IV confers coherent, multi-level benefits that are mechanistically aligned with the pathophysiology of PMOP. AS-IV treatment effectively attenuated the high bone turnover phenotype as indicated by reduced serum levels of the resorption marker CTX-I and a partial normalization of the formation marker OCN and parathyroid hormone (PTH). Histological analysis revealed improved trabecular thickness and connectivity. These structural improvements were directionally consistent with, though not statistically significant in, micro-CT analyses, which showed trends toward increased bone mineral density (BMD), bone volume fraction (BV/TV), and trabecular number (Tb.N), and decreased trabecular separation (Tb. Sp). Unbiased transcriptomics converged on osteoimmune and Wnt programs and highlighted eight hub genes. Molecular docking simulations substantiated these findings by predicting favorable binding affinities between AS-IV and several of these prioritized targets. Collectively, these findings propose that AS-IV addresses interacting axes of estrogen-deficiency–driven osteoimmune activation and impaired osteoblastogenesis that underpin PMOP (23, 24).

The correcting high bone turnover signatures is pathophysiologically relevant in PMOP: lowering CTX-I reflects suppression of osteoclast-mediated type I collagen degradation, partial normalization of OCN indicates restoration of balanced remodeling, and attenuation of PTH helps curb high-turnover bone loss and supports mineralization—consistent with consensus frameworks for turnover markers in PMOP (25–27). These biochemical markers are commonly used in the evaluation of osteoporosis and are reflective of systemic changes in bone remodeling, reinforcing the therapeutic potential of AS-IV in addressing PMOP.

The antioxidant effects of AS-IV are also consistent with the recognized role of oxidative stress in PMOP pathogenesis. The observed increase in superoxide dismutase (SOD) activity reflects restored redox homeostasis and supports osteoblast function, whereas lower malondialdehyde (MDA) indicates reduced lipid-peroxidation damage; in OVX rats, interventions that increase SOD and decrease MDA consistently attenuate bone loss (28–31). These findings confirm that AS-IV modulates oxidative stress as a critical mechanism for protecting against PMOP.

Endpoint changes on micro-CT were directionally consistent with histology but did not reach statistical significance (32). This lack of significance likely reflects several methodological factors inherent in small-animal studies. First, the sample size (*n* = 3/group) limits the ability to detect moderate effects unless the magnitude of change is large and variability is low. Second, tissue-level connectivity and surface remodeling can precede volumetric accrual detectable by micro-CT, so early histological gains may not yet translate into significant morphometric separation (33). Third, technical factors including VOI selection, voxel resolution, segmentation thresholds, and partial-volume effects can materially influence detectable effect sizes in trabecular bone analysis (32–35). Therefore, the non-significant micro-CT differences, alongside clear histological improvement, likely reflect kinetic and methodological ceilings rather than an absence of biological effect (32, 33). While these micro-CT findings remain exploratory, they do not diminish the study’s core contributions: the biochemical, oxidative stress, and transcriptomic data provide a strong foundation for understanding AS-IV’s mechanisms and designing future confirmatory studies with optimized parameters and larger cohorts.

Transcriptomics converged on osteoimmune and Wnt-related programs and, together with docking, prioritized eight hub genes: IL22RA2, PTGS2, WNT1, IGFBP2, GZMB, LEP, GFAP, WNT10A. IL22RA2 (IL-22 binding protein) neutralizes IL-22 with high affinity and can restrain IL-22–driven RANKL induction/osteoclastogenesis, aligning with reduced resorption (36). PTGS2 (COX-2) couples inflammatory tone to bone remodeling and fracture repair; context-appropriate modulation can curb excessive resorption while preserving physiologic healing (37). WNT1 is a dosage-sensitive anabolic determinant of bone mass in humans; engagement is compatible with improved trabecular connectivity (38). IGFBP-2 shapes IGF bioavailability and can modify formation dynamics, offering a conduit to improve the formation–resorption balance (39, 40). Immune cytotoxic programs that involve granzyme B (GZMB) promote osteoclastogenesis and bone destruction in arthritis models; dampening this arm would favor remodeling balance (41). Leptin signaling via a hypothalamic–sympathetic relay suppresses osteogenesis and alters remodeling; mitigating leptin-linked pathways could counter the high-turnover phenotype of PMOP (42, 43). GFAP marks non-myelinating Schwann cells that maintain marrow niches and contribute to neural regulation of remodeling, increasingly implicated in estrogen-deficient bone (44). Finally, WNT10A biases mesenchymal fate toward osteoblastogenesis and away from adipogenesis, providing a route to favor bone formation over marrow adiposity after estrogen loss (45).

Structural plausibility is supported by docking with a validated engine (AutoDock Vina), which yielded favorable binding energies for several hubs and complements the biological readouts (46). Prior experimental work with AS-IV—enhancing osteogenesis–angiogenesis coupling and restraining osteoclastogenesis—further supports this integrated interpretation in OVX/PMOP settings (47).

The lack of significant recovery in *β*-catenin suggests that AS-IV does not directly restore canonical Wnt/β-catenin signaling in this model. While β-catenin is a key marker of canonical Wnt signaling, our findings indicate that AS-IV likely modulates Wnt signaling through non-canonical pathways, particularly via Wnt1, which was significantly upregulated in treated animals. Wnt1, acting as an upstream ligand, may activate non-canonical Wnt signaling independent of β-catenin stabilization or nuclear translocation (48). As such, AS-IV appears to modulate key components of the Wnt pathway through non-canonical mechanisms. Additionally, the discrepancy between Lep transcript levels and protein expression suggests complex regulatory mechanisms, such as post-transcriptional control, affecting protein secretion and retention (49, 50). Although Lep mRNA was upregulated in OVX rats, protein expression did not align, possibly due to secretion dynamics or cell-type specific retention. Future studies integrating circulating leptin and cell-specific analyses are needed to clarify this relationship. Furthermore, while Igfbp2 exhibited changes at the transcriptomic level, no corresponding protein modulation was observed in the femoral trabecular region, suggesting that Igfbp2 may not be a primary target of AS-IV in this model or that its role in bone remodeling involves more complex regulatory mechanisms not captured by the immunofluorescence assay (39). These nuanced findings do not diminish the validity of our transcriptomic data but rather underscore the multi-layered regulation of bone-active pathways and highlight the necessity of future functional studies—including genetic perturbation, circulating biomarker measurements, and cell-type-specific analyses—to establish causal relationships.

Collectively, our findings suggest that AS-IV mitigates OVX-induced bone loss by converging on osteoimmune restraint (IL-22/IL22RA2, GZMB, PTGS2), anabolic Wnt drive (WNT1/WNT10A), endocrine–metabolic tuning (IGFBP-2/IGF axis), and neural–marrow support (GFAP). The cross-talk among these axes is central to PMOP pathophysiology, and the alignment of docking-nominated hubs with coherent marker, histology, and imaging responses positions AS-IV as a mechanistically grounded candidate for PMOP management. Conceptually, this work advances a multi-target perspective on AS-IV in bone remodeling by integrating imaging, biochemical, and network-level transcriptomic signals, thereby generating testable hypotheses for target engagement and pathway modulation beyond single-pathway explanations.

As an exploratory investigation, this study has several limitations. The modest sample size (n = 3/group for micro-CT) limited statistical power for structural endpoints, and the absence of functional perturbation experiments means our findings remain correlative and hypothesis-generating rather than confirmatory of causal mechanisms. To advance from integrative signals to causal inference and translational readiness, future work should strengthen study design and mechanistic resolution through three priority areas: (i) functional validation using genetic or pharmacological manipulation of prioritized pathways; (ii) enhanced study design with prospectively powered cohorts, extended treatment duration, time-course sampling, and rigorous randomization/blinding; and (iii) higher-resolution technologies such as single-cell or spatial transcriptomics to deconvolve cell-type-specific responses within the bone marrow niche. For generalizability and translation, replication in glucocorticoid-induced and aging-related osteoporosis models is recommended, pairing micro-CT with finite-element analysis to estimate bone strength and exploring combination regimens with standard-of-care therapies. Collectively, these efforts will convert associative multi-omics evidence into mechanism-anchored, clinically meaningful advances.

In conclusion, by integrating multi-level data, this study establishes AS-IV as a mechanistically grounded candidate for PMOP management. It provides a rich, testable framework linking osteoimmune modulation, Wnt-related signaling, and redox balance to skeletal protection, thereby laying a strong foundation for subsequent causal and translational research.

## 5 Conclusion

In conclusion, this multi-level preclinical investigation demonstrates that Astragaloside IV (AS-IV) exerts coordinated skeletal protection in the OVX-induced osteoporosis model through a multi-target mechanism converging on osteoimmune modulation and redox balance. AS-IV attenuated the high-turnover phenotype by favorably regulating bone turnover markers (CTX-I, OCN, PTH) and systemic oxidative stress (SOD, MDA), which translated into qualitative improvements in trabecular architecture observed histologically. Although micro-CT analysis did not reach statistical significance—likely reflecting methodological constraints of this exploratory study—the directional consistency across biochemical, histological, and imaging modalities supports a genuine biological effect warranting further investigation with optimized study designs.

Integrating transcriptomic profiling with molecular docking nominated osteoimmune (e.g., Il22ra2, Ptgs2) and Wnt-related (e.g., Wnt1) hubs as candidate mediators of AS-IV’s effects. Tissue-level validation further revealed partial normalization of OVX-induced NF-κB p65 activation and Wnt1 suppression, while the absence of significant changes in *β*-catenin and Ptgs2 protein highlights the pathway-specific and potentially non-canonical nature of AS-IV’s mechanism. These nuanced findings do not diminish the validity of our multi-omic data but rather underscore the complex, multi-layered regulation of bone-active pathways.

Collectively, the convergence of evidence across serum biomarkers, bone architecture, transcriptomics, and protein validation establishes AS-IV as a mechanistically grounded, nutraceutical-inspired candidate for postmenopausal osteoporosis management. By generating a robust, testable framework linking inflammatory restraint, redox homeostasis, and osteogenic cues to skeletal protection, this study provides a strong foundation for future causal validation studies with larger cohorts, extended treatment windows, and functional perturbation assays.

## Data Availability

The RNA sequencing data generated in this study have been deposited in the NCBI GEO database under accession number GSE326719. The data are publicly available at https://www.ncbi.nlm.nih.gov/geo/query/acc.cgi?acc=GSE326719.

## Footnotes

1
https://cn.string-db.org/

2
https://www.microbiome.cn/

3
https://pubchem.ncbi.nlm.nih.gov/

4
https://www.rcsb.org/

5
https://alphafold.com/

## References

[1] Møller AMJ Delaissé J-M Olesen JB Madsen JS Canto LM Bechmann T . Aging and menopause reprogram osteoclast precursors for aggressive bone resorption. Bone Res. (2020) 8:27. doi: 10.1038/s41413-020-0102-7, 32637185 PMC7329827

[2] Eastell R O’Neill TW Hofbauer LC Langdahl B Reid IR Gold DT . Postmenopausal osteoporosis. Nat Rev Dis Primers. (2016) 2:16069. doi: 10.1038/nrdp.2016.69, 27681935

[3] Yong E-L Logan S. Menopausal osteoporosis: screening, prevention and treatment. Singapore Med J. (2021) 62:159–66. doi: 10.11622/smedj.2021036, 33948669 PMC8801823

[4] Yao Y Cai X Chen Y Zhang M Zheng C. Estrogen deficiency-mediated osteoimmunity in postmenopausal osteoporosis. Med Res Rev. (2025) 45:561–75. doi: 10.1002/med.22081, 39234932

[5] Bonucci E Ballanti P. Osteoporosis-bone remodeling and animal models. Toxicol Pathol. (2014) 42:957–69. doi: 10.1177/0192623313512428, 24285673

[6] Gosset A Pouillès J-M Trémollieres F. Menopausal hormone therapy for the management of osteoporosis. Best Pract Res Clin Endocrinol Metab. (2021) 35:101551. doi: 10.1016/j.beem.2021.101551, 34119418

[7] Bluming AZ. Hormone replacement therapy after breast cancer: it is time. Cancer J. (2022) 28:183–190. doi: 10.1097/PPO.0000000000000595 35594465

[8] Sjögren LL Mørch LS Løkkegaard E. Hormone replacement therapy and the risk of endometrial cancer: a systematic review. Maturitas. (2016) 91:25–35. doi: 10.1016/j.maturitas.2016.05.013, 27451318

[9] Rossouw JE Anderson GL Prentice RL LaCroix AZ Kooperberg C Stefanick ML . Risks and benefits of estrogen plus progestin in healthy postmenopausal women: principal results from the women’s health initiative randomized controlled trial. JAMA. (2002) 288:321–33. doi: 10.1001/jama.288.3.321, 12117397

[10] Nelson HD Humphrey LL Nygren P Teutsch SM Allan JD. Postmenopausal hormone replacement therapy: scientific review. JAMA. (2002) 288:872–81. doi: 10.1001/jama.288.7.872, 12186605

[11] Brech GC de Paula TS Fedele TA Dias AS Soares-Júnior JM Bordalo-Rodrigues M . Response to fatigue observed through magnetic resonance imaging on the quadriceps muscle in postmenopausal women. Clinics (Sao Paulo). (2020) 75:e1768. doi: 10.6061/clinics/2020/e1768, 32609225 PMC7314579

[12] Shim K-S Hwang Y-H Jang S-A Kim T Ha H. Water extract of *Lysimachia christinae* inhibits trabecular bone loss and fat accumulation in ovariectomized mice. Nutrients. (2020) 12:1927. doi: 10.3390/nu12071927, 32610585 PMC7399897

[13] Zhang J Wu C Gao L Du G Qin X. Astragaloside IV derived from *Astragalus membranaceus*: a research review on the pharmacological effects. Adv Pharmacol. (2020) 87:89–112. doi: 10.1016/bs.apha.2019.08.002, 32089240

[14] Auyeung KK Han Q-B Ko JK. *Astragalus membranaceus*: a review of its protection against inflammation and gastrointestinal cancers. Am J Chin Med. (2016) 44:1–22. doi: 10.1142/S0192415X16500014, 26916911

[15] Fu J Wang Z Huang L Zheng S Wang D Chen S . Review of the botanical characteristics, phytochemistry, and pharmacology of *Astragalus membranaceus* (Huangqi). Phytother Res. (2014) 28:1275–83. doi: 10.1002/ptr.5188, 25087616

[16] Liu P Zhao H Luo Y. Anti-aging implications of *Astragalus membranaceus* (Huangqi): a well-known Chinese tonic. Aging Dis. (2017) 8:868–86. doi: 10.14336/AD.2017.0816, 29344421 PMC5758356

[17] Wang S Peng Y Zhuang Y Wang N Jin J Zhan Z. Purification, structural analysis and cardio-protective activity of polysaccharides from Radix astragali. Molecules. (2023) 28:4167. doi: 10.3390/molecules28104167, 37241906 PMC10222213

[18] Wang F Qian H Kong L Wang W Wang X Xu Z . Accelerated bone regeneration by Astragaloside IV through stimulating the coupling of osteogenesis and angiogenesis. Int J Biol Sci. (2021) 17:1821–36. doi: 10.7150/ijbs.57681, 33994865 PMC8120474

[19] Wang W-L Sheu S-Y Chen Y-S Kao S-T Fu Y-T Kuo T-F . Evaluating the bone tissue regeneration capability of the Chinese herbal decoction Danggui Buxue tang from a molecular biology perspective. Biomed Res Int. (2014) 2014:853234. doi: 10.1155/2014/853234, 25295277 PMC4176646

[20] Wang H Huang Z Chen G Li Y Liu Y Gu H . Astragaloside IV alleviated bone loss in mice with ovariectomy-induced osteoporosis via modulating gut microbiota and fecal metabolism. Front Pharmacol. (2025) 16:1548491. doi: 10.3389/fphar.2025.1548491, 40248089 PMC12003300

[21] Zhang X Li M Wang H. Astragaloside IV alleviates the myocardial damage induced by lipopolysaccharide via the toll-like receptor 4 (TLR4)/nuclear factor kappa B (NF-κB)/proliferator-activated receptor α (PPARα) Signaling pathway. Med Sci Monit. (2019) 25:7158–68. doi: 10.12659/MSM.916030, 31545785 PMC6775796

[22] Luo C Ye Y Lv A Zuo W Yang Y Jiang C . The impact of Astragaloside IV on the inflammatory response and gut microbiota in cases of acute lung injury is examined through the utilization of the PI3K/AKT/mTOR pathway. PLoS One. (2024) 19:e0305058. doi: 10.1371/journal.pone.0305058, 38954702 PMC11218977

[23] Weitzmann MN Pacifici R. Estrogen deficiency and bone loss: an inflammatory tale. J Clin Invest. (2006) 116:1186–94. doi: 10.1172/JCI28550, 16670759 PMC1451218

[24] Takayanagi H. Osteoimmunology: shared mechanisms and crosstalk between the immune and bone systems. Nat Rev Immunol. (2007) 7:292–304. doi: 10.1038/nri2062, 17380158

[25] Eastell R Szulc P. Use of bone turnover markers in postmenopausal osteoporosis. Lancet Diabetes Endocrinol. (2017) 5:908–23. doi: 10.1016/S2213-8587(17)30184-5, 28689768

[26] Vasikaran S Eastell R Bruyère O Foldes AJ Garnero P Griesmacher A . Markers of bone turnover for the prediction of fracture risk and monitoring of osteoporosis treatment: a need for international reference standards. Osteoporos Int. (2011) 22:391–420. doi: 10.1007/s00198-010-1501-1, 21184054

[27] Lips P. Vitamin D deficiency and secondary hyperparathyroidism in the elderly: consequences for bone loss and fractures and therapeutic implications. Endocr Rev. (2001) 22:477–501. doi: 10.1210/edrv.22.4.0437, 11493580

[28] Iantomasi T Romagnoli C Palmini G Donati S Falsetti I Miglietta F . Oxidative stress and inflammation in osteoporosis: molecular mechanisms involved and the relationship with microRNAs. Int J Mol Sci. (2023) 24:3772. doi: 10.3390/ijms24043772, 36835184 PMC9963528

[29] Marcucci G Domazetovic V Nediani C Ruzzolini J Favre C Brandi ML. Oxidative stress and natural antioxidants in osteoporosis: novel preventive and therapeutic approaches. Antioxidants (Basel). (2023) 12:373. doi: 10.3390/antiox12020373, 36829932 PMC9952369

[30] Guo J-D Li L Shi Y-M Wang H-D Hou S-X. Hydrogen water consumption prevents osteopenia in ovariectomized rats. Br J Pharmacol. (2013) 168:1412–20. doi: 10.1111/bph.12036, 23121335 PMC3596646

[31] Dong X-L Li C-M Cao S-S Zhou L-P Wong M-S. A high-saturated-fat, high-sucrose diet aggravates bone loss in ovariectomized female rats. J Nutr. (2016) 146:1172–9. doi: 10.3945/jn.115.225474, 27099231

[32] Bouxsein ML Boyd SK Christiansen BA Guldberg RE Jepsen KJ Müller R. Guidelines for assessment of bone microstructure in rodents using micro-computed tomography. J Bone Miner Res. (2010) 25:1468–86. doi: 10.1002/jbmr.141, 20533309

[33] Campbell GM Sophocleous A. Quantitative analysis of bone and soft tissue by micro-computed tomography: applications to ex vivo and in vivo studies. Bonekey Rep. (2014) 3:564. doi: 10.1038/bonekey.2014.59, 25184037 PMC4140449

[34] Odgaard A. Three-dimensional methods for quantification of cancellous bone architecture. Bone. (1997) 20:315–28. doi: 10.1016/s8756-3282(97)00007-0, 9108351

[35] Hildebrand TOR Rüegsegger P. Quantification of bone microarchitecture with the structure model index. Comput Methods Biomech Biomed Engin. (1997) 1:15–23. doi: 10.1080/01495739708936692, 11264794

[36] Zenewicz LA. IL-22 binding protein (IL-22BP) in the regulation of IL-22 biology. Front Immunol. (2021) 12:766586. doi: 10.3389/fimmu.2021.766586, 34868019 PMC8634938

[37] Simon AM Manigrasso MB O’Connor JP. Cyclo-oxygenase 2 function is essential for bone fracture healing. J Bone Miner Res. (2002) 17:963–76. doi: 10.1359/jbmr.2002.17.6.963, 12054171

[38] Laine CM Joeng KS Campeau PM Kiviranta R Tarkkonen K Grover M . WNT1 mutations in early-onset osteoporosis and osteogenesis imperfecta. N Engl J Med. (2013) 368:1809–16. doi: 10.1056/NEJMoa1215458, 23656646 PMC3709450

[39] Conover. Insulin-like growth factor-binding proteins and bone metabolism. Am J Physiol Endocrinol Metab (2008) 294, E10–E14. doi:doi: 10.1152/ajpendo.00648.2007 18003717

[40] Xi G Wai C Rosen CJ Clemmons DR. A peptide containing the receptor binding site of insulin-like growth factor binding protein-2 enhances bone mass in ovariectomized rats. Bone Res. (2018) 6:23. doi: 10.1038/s41413-018-0024-9, 30109160 PMC6089876

[41] Söderström K Stein E Colmenero P Purath U Müller-Ladner U de Matos CT . Natural killer cells trigger osteoclastogenesis and bone destruction in arthritis. Proc Natl Acad Sci USA. (2010) 107:13028–33. doi: 10.1073/pnas.1000546107, 20615964 PMC2919936

[42] Ducy P Amling M Takeda S Priemel M Schilling AF Beil FT . Leptin inhibits bone formation through a hypothalamic relay: a central control of bone mass. Cell. (2000) 100:197–207. doi: 10.1016/s0092-8674(00)81558-5, 10660043

[43] Karsenty G Oury F. The central regulation of bone mass, the first link between bone remodeling and energy metabolism. J Clin Endocrinol Metab. (2010) 95:4795–801. doi: 10.1210/jc.2010-1030, 21051575

[44] Yamazaki S Ema H Karlsson G Yamaguchi T Miyoshi H Shioda S . Nonmyelinating Schwann cells maintain hematopoietic stem cell hibernation in the bone marrow niche. Cell. (2011) 147:1146–58. doi: 10.1016/j.cell.2011.09.053, 22118468

[45] Cawthorn WP Bree AJ Yao Y Du B Hemati N Martinez-Santibañez G . Wnt6, Wnt10a and Wnt10b inhibit adipogenesis and stimulate osteoblastogenesis through a β-catenin-dependent mechanism. Bone. (2012) 50:477–89. doi: 10.1016/j.bone.2011.08.010, 21872687 PMC3261372

[46] Trott O Olson AJ. AutoDock Vina: improving the speed and accuracy of docking with a new scoring function, efficient optimization, and multithreading. J Comput Chem. (2010) 31:455–61. doi: 10.1002/jcc.21334, 19499576 PMC3041641

[47] Liang Y Chen B Liang D Quan X Gu R Meng Z . Pharmacological effects of Astragaloside IV: a review. Molecules. (2023) 28:6118. doi: 10.3390/molecules28166118, 37630371 PMC10458270

[48] Liang J Pan Y Yang J Zeng D Li J. WNT signaling in cancer: molecular mechanisms and potential therapies. Mol Biomed. (2025) 6:83. doi: 10.1186/s43556-025-00327-x, 41120801 PMC12540965

[49] Choi S Kang J-G Tran YTH Jeong S-H Park K-Y Shin H . Hippo–YAP/TAZ signalling coordinates adipose plasticity and energy balance by uncoupling leptin expression from fat mass. Nat Metab. (2024) 6:847–60. doi: 10.1038/s42255-024-01045-4, 38811804 PMC11136666

[50] Chen J Xu J Gou L Zhu Y Zhong W Guo H . Integrating transcriptomic and proteomic data for a comprehensive molecular perspective on the association between sarcopenia and osteoporosis. Arch Gerontol Geriatr. (2024) 125:105486. doi: 10.1016/j.archger.2024.105486, 38761527

