# Supplementary material for: Astragaloside IV modulates oxidative stress and osteoimmune–Wnt signaling in ovariectomized rats: an integrated study of RNA sequencing, molecular docking, and experimental validation
Source: Front Nutr. 2026 Apr 13;13:1785452. doi: 10.3389/fnut.2026.1785452 (PMC13111271; doi:10.3389/fnut.2026.1785452)
Supplement: Supplementary file 1 [file Supplementary_File_1.docx]

Supplementary Material

# Supplementary Figures

**
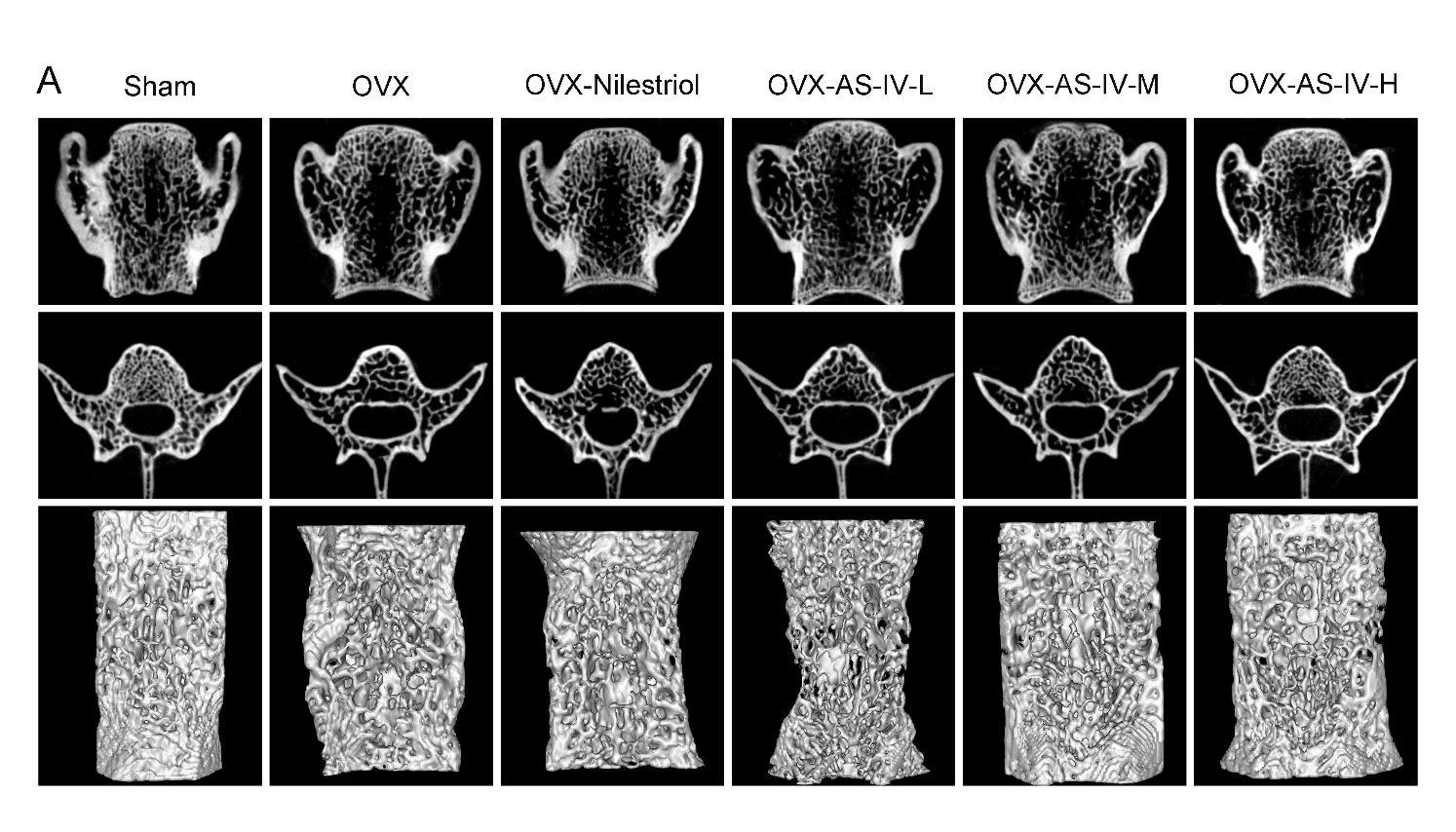
**

**
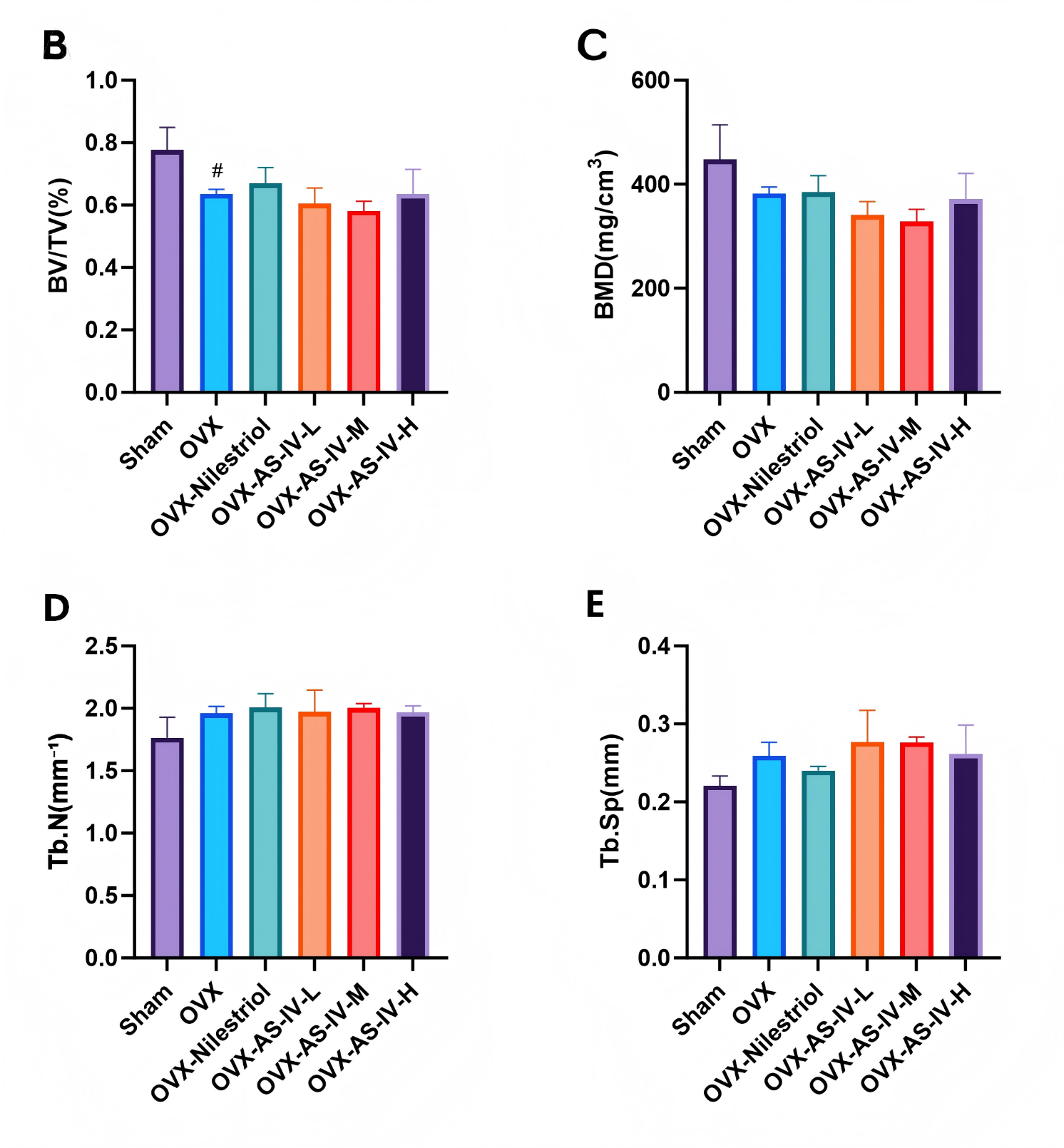
**

**Supplementary Figure 1.** Micro-CT scans and 3D reconstruction images and data of rat vertebrae tissue.(A) Micro-CT Images of the Vertebrae: 2D Coronal, 2D Cross-Sectional, and 3D Trabecular Views.(B) bone volume/ total volume (BV/TV, %).(C) bone mineral density (BMD, mg/cm3 ).(D) trabecular number (Tb.N, 1/mm).(E) trabecular separation (Tb.Sp, mm). # p < 0.05 vs. sham group.


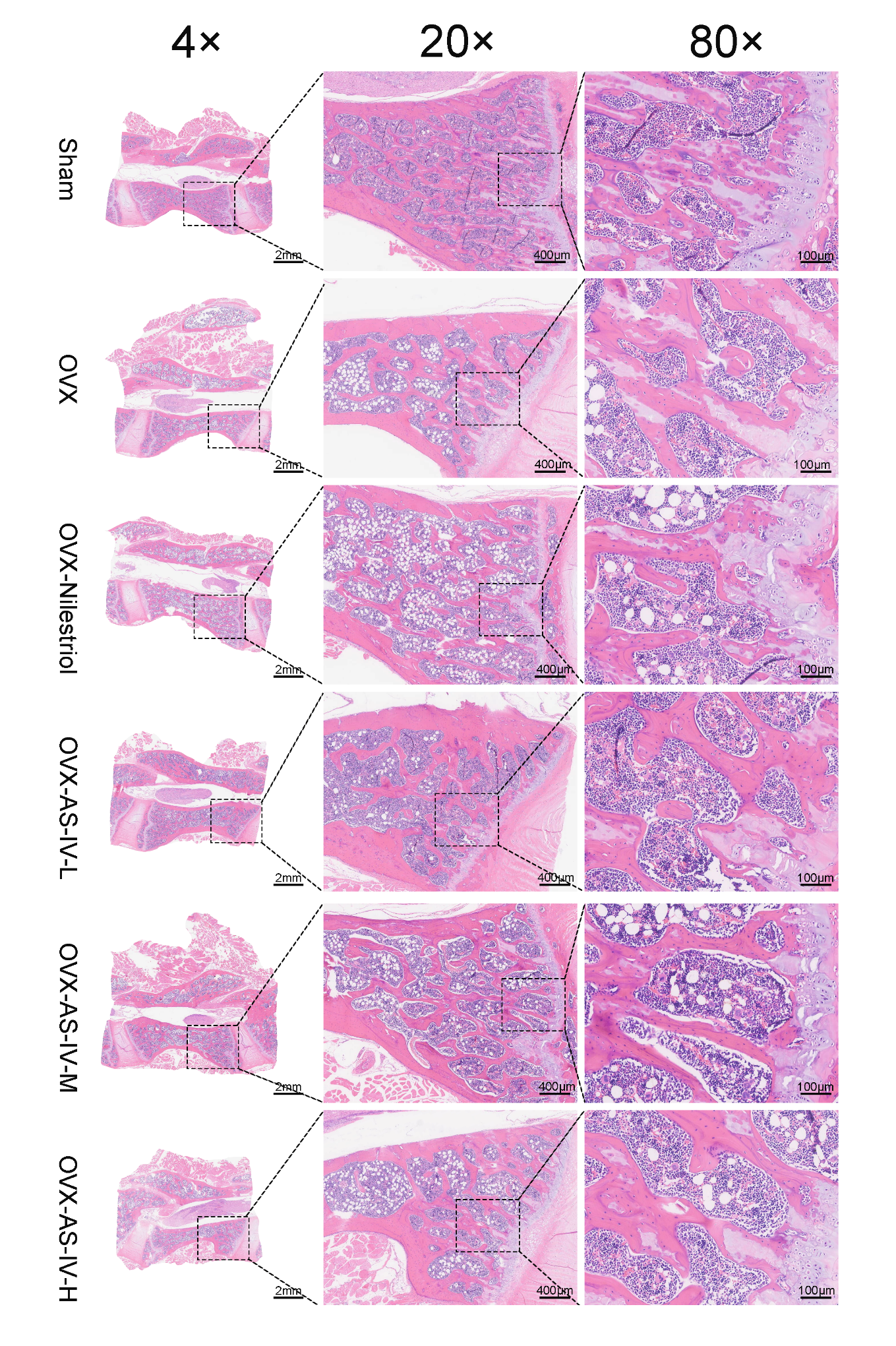


**Supplementary Figure 2.** H&E histology of decalcified paraffin sections from rat vertebral trabecular bone shown as a three-magnification composite (4×, 20×, 80×; left-to-right within each group). Scale bars as indicated.
